# European maize genomes highlight intraspecies variation in repeat and gene content

**DOI:** 10.1038/s41588-020-0671-9

**Published:** 2020-07-27

**Authors:** Georg Haberer, Nadia Kamal, Eva Bauer, Heidrun Gundlach, Iris Fischer, Michael A. Seidel, Manuel Spannagl, Caroline Marcon, Alevtina Ruban, Claude Urbany, Adnane Nemri, Frank Hochholdinger, Milena Ouzunova, Andreas Houben, Chris-Carolin Schön, Klaus F. X. Mayer

**Affiliations:** 1 Plant Genome and Systems Biology, Helmholtz Center Munich, Neuherberg, Germany; 2 Plant Breeding, School of Life Sciences, Technical University Munich, Munich, Germany; 3 Crop Functional Genomics, Institute for Crop Science and Resource Conservation, University of Bonn, Bonn, Germany; 4 Leibniz Institute of Plant Genetics and Crop Plant Research, Seeland, Germany; 5 KWS SAAT SE, Einbeck, Germany; 6 School of Life Sciences, Technical University Munich, Munich, Germany

**Keywords:** Plant genetics, Genomics, Plant breeding

## Abstract

The diversity of maize (*Zea mays*) is the backbone of modern heterotic patterns and hybrid breeding. Historically, US farmers exploited this variability to establish today’s highly productive Corn Belt inbred lines from blends of dent and flint germplasm pools. Here, we report de novo genome sequences of four European flint lines assembled to pseudomolecules with scaffold N50 ranging from 6.1 to 10.4 Mb. Comparative analyses with two US Corn Belt lines explains the pronounced differences between both germplasms. While overall syntenic order and consolidated gene annotations reveal only moderate pangenomic differences, whole-genome alignments delineating the core and dispensable genome, and the analysis of heterochromatic knobs and orthologous long terminal repeat retrotransposons unveil the dynamics of the maize genome. The high-quality genome sequences of the flint pool complement the maize pangenome and provide an important tool to study maize improvement at a genome scale and to enhance modern hybrid breeding.

## Abstract (web summary)

De novo genome assemblies of four European flint maize lines and comparison with two US Corn Belt genomes provide insights into the dynamics of intraspecies variation in repeat and gene content in maize genomes.

## Main

Since its domestication ~10,000 years ago by Native Americans, maize (*Zea mays* ssp. *mays*) has become one of the most important sources for human nutrition and animal feeding. Extensive variation in landraces and breeding germplasm such as dent or flint corns underpins the enormous phenotypic and genetic diversity of maize^1–3^. Today, US hybrids produced from inbred lines of different heterotic groups; for example, stiff stalk and nonstiff stalk^4^ are highly productive and agriculturally important worldwide. These US Corn Belt dents resulted from crosses in the nineteenth century between Southern dent lines introduced to the United States from Mexico and Northern flints (NF) which were the predominant germplasm in the pre-Columbian era grown by the Native Americans^5,6^. Historical records and genetic and molecular data strongly indicate that NFs originated from Native American populations of Southwestern America and the Great Plains^2,6,7^. Improvement by early farmers and adaptation to cooler climate and different photoperiods extended NF growth as far north as Southern Canada. After the colonization of the New World, maize was spread to Europe both from the Caribbean islands and Northeast United States^8^. The early maturing and cold-tolerant flints were key to a successful maize cultivation in temperate regions of Europe. Consequently, NF germplasm still makes a major contribution to modern European maize breeding material while Corn Belt dent genomes contain on average one-quarter from their blending with NFs^2,6^.

The complete genome sequence of the dent line B73 and its continuous updates provide a high-quality reference for maize breeding, genetic and genomic research^9,10^. However, very high diversity at both the sequence and genic level has been reported between maize inbred lines for targeted regions and large-scale comparisons^3,11–14^. Hence, the B73 reference sequence captures only a portion of the maize pangenome. To overcome these limitations, a draft genome of the Iodent line PH207 (ref. ^15^) and—more recently—reference sequences of SK, Mo17 and W22, a tropical and two US dent lines, have been released^16–18^. Worldwide, many hybrid breeding programs focus on dent germplasm, whereas breeding programs in cooler regions of Central Europe exploit heterotic effects between dent and flint lines. Several studies have shown a clear differentiation of the North American and Northern European flint germplasm from the rest of the world^19^. While reference-quality sequences exist for several dent inbred lines, the flint pool is still underexploited. To date, only one fragmented draft assembly with an N50 ~13.9 kilobases (kb) of one flint line, F2, is available covering ~65% of the estimated genome size^20^. To better understand the extent and quality of genomic differences between the flint and dent germplasm pools we generated de novo high-quality sequences for four flint inbred lines representing important ancestors of maize hybrid breeding in Central Europe^21^ and contrasted in this study their gene and repeat contents to two US Corn Belt dents.

## Results

### Whole-genome sequencing

**Extended Data Fig. 1:**
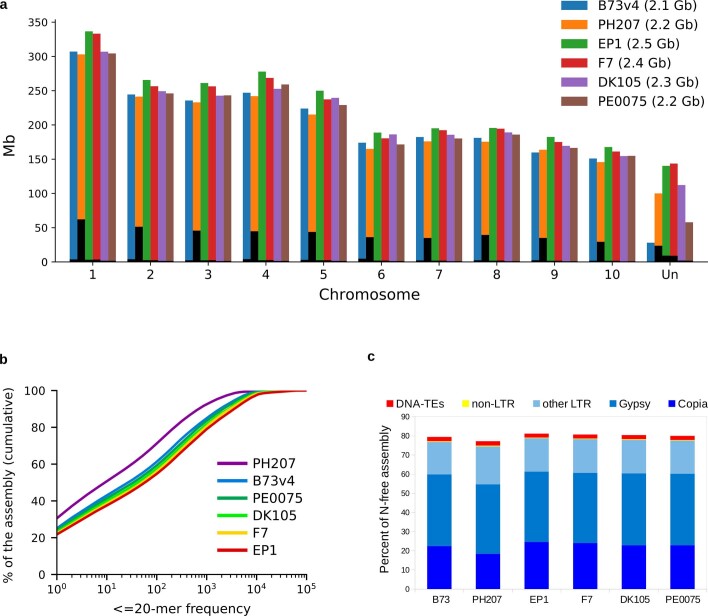
Assembly sizes and overall repeat content of 8 maize lines. **a**, Chromosome sizes and N-content (black bars), **b**, Basic repetitivity in form of 20mer frequencies. Cumulative plot, lower curves represent higher repetitivity e.g. 50% of the PH207 assembly consists of 20mers occurring < =10 times, for the other lines this value is only around 40%. **c**, Transposon content.

**Extended Data Fig. 2:**
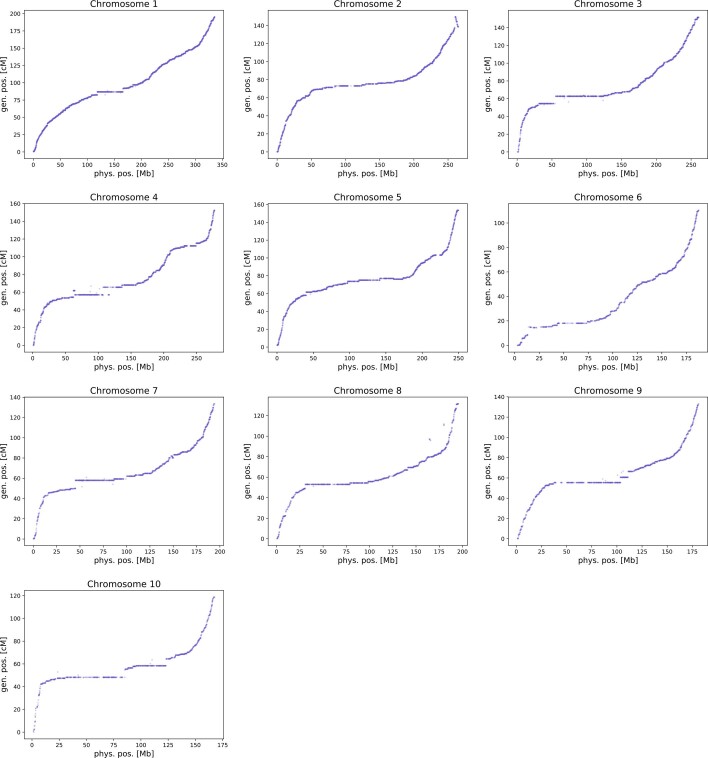
Genetic versus physical distances for all ten chromosomes of the EP1xPH207 cross. X-axis: physical position on the respective EP1 chromosome in [Mb], Y-axis: genetic position in centiMorgan [cM].

To access the genomic landscape of European flint maize, we assembled four flint lines to reference genome quality. Three lines are important founders of European breeding programs and represent distinct flint germplasm sources such as populations Lacaune (F7, Southern France), Lizargarate (EP1, Northern Spain) and Gelber Badischer Landmais (DK105, Southern Germany). The fourth sample, PE0075, is a doubled-haploid line derived from the Petkuser Ferdinand Rot population, a landrace from Northern Germany. We generated Illumina paired-end and mate-pair sequences equivalent to ~220–320× coverage (Supplementary Table 1) and assembled the reads to scaffolds and pseudomolecules using the DeNovoMagic pipeline^15,16,22^. Total assembly sizes of the pseudochromosomes range from 2.14 to 2.32 megabases (Mb) and amount to 92.9–100.5% of the genome sizes estimated by *K*-mer analysis (Table 1 and Supplementary Fig. 1). Most (94.3–97.3%) contig and scaffold sequences were integrated into ten pseudochromosomes (Table 1, Supplementary Table 2 and Extended Data Fig. 1a). A genetic map generated from an F_2_ mapping population of an EP1 × PH207 cross demonstrated a high consensus between genetic and physical map corroborating quality and contiguity of the maize assemblies (Extended Data Fig. 2). Sequence accuracy was high with less than one erroneous base per 100 kb (Supplementary Table 3) and remapping genomic reads using SQUAT^23^ reported <2% poorly mapped reads. Complete BUSCO^24^ genes totaled >95%, strongly supporting high coverage of the gene space while only ~1.5% and 0.8–2.4% of the BUSCO Liliopsidae set were absent or fragmented in each of the four assemblies on average, respectively (Supplementary Table 2). In addition, the long terminal repeat (LTR) assembly index^25^, a measure for the correct reconstruction of the repetitive transposon space, supports the reference quality of our four flint assemblies (Supplementary Table 4).

**Table 1:** Genome statistics of maize dent and flint lines used in this study

	*Zea mays* ‘flint’ lines				*Zea mays* ‘dent’ lines	
Line	EP1	F7	DK105	PE0075	B73 (v.4)	PH207
Assembly size (Mb)	2,455.3	2,392.8	2,288.2	2,198.5	2,135.1	2,156.2
Chromosome (Mb)	2,321.0	2,255.5	2,176.1	2,140.7	2,106.3	2,060.3
Scaffolds (*n*)	60,567	62,610	797	972	267	4,3291
Scaffold N50 (Mb)	6.13	9.48	10.39	8.64	ND	ND
Genes (*n*)	47,174	48,068	46,697	46,742	48,003	46,207
Orthologs	45,630	45,932	45,697	45,824	45,928	43,777
Class I repeats (% of assembly)	79.1	78.6	78.3	77.8	77.2	74.9
Class II repeats (% of assembly)	2.03	2.07	2.11	2.16	2.28	2.29

The table summarizes total assembly size, total size of assembled chromosomes, number of pseudochromosomes including unanchored scaffolds, N50 of scaffolds before pseudochromosome generation, total size of undefined sequence in the genome, number of genes (including low-confidence genes; see Main text and Supplementary Information), orthologs (defined as bidirectional best Blast hits) and total percentages of class I and class II repeats. ND, not determined.

#### Pan-gene variation among flint and dent is moderate

**Fig. 1:**
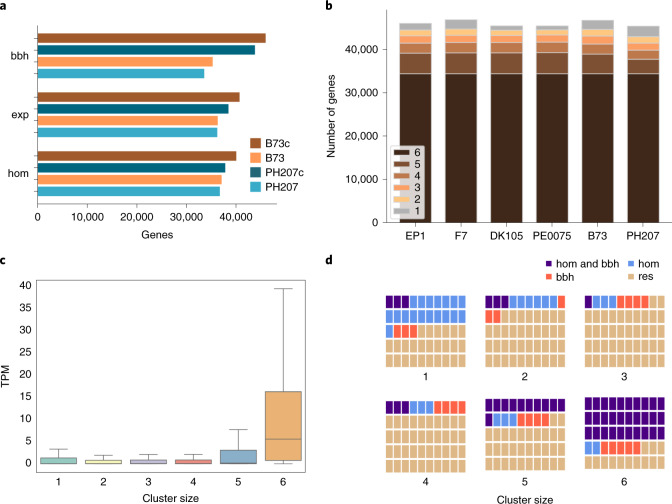
Gene consolidation and characteristics. **a**, Comparison of reciprocal best blast orthologs (bbh), genes with a TPM ≥0.1 (exp) and angiosperm proteins (hom) between the consolidated (B73c and PH207c) and initial B73 and PH207 gene models. **b**, The number of genes per line that are either singletons (label 1) or syntenic between two and six lines (labels 2–6). **c**, Expression levels (TPM) of EP1 and F7 genes graded by size of their orthologous clusters. Genes either are singletons (cluster size 1) or have orthologs in one to five maize lines (cluster sizes 2–6). Syntelogs of orthologous clusters comprising 2–4 lines show very low median expression levels (TPM ~0.06). **d**, Illustration per cluster size of the proportion of orthologous groups with support by bbh and homology (purple), only bbh (red), only homology (hom, blue) and residual clusters (res, tan) matching neither the homology nor the bbh criterion. To record homology, a minimum alignment length of 80% of both maize and angiosperm protein was required in at least three species. The high conservation observed in cluster size 1 reflects, in part, enrichment of tandemly repeated genes duplicated from orthologs of clusters present in five or six lines.

To assess genic presence–absence variations (PAVs) in the six maize lines we used a three-layer gene prediction pipeline. For F7 and EP1 we predicted protein-coding genes as consensus models using protein homologies of known monocotyledonous protein sequences and a broad spectrum of transcriptome evidences from F7 and EP1 RNA-seq data (Supplementary Table 5). Subsequently, cross-mapping of the EP1 and F7 models as well as annotations of B73 (v.4) and PH207 (v.1.1) complemented the gene sets of all six maize lines including PE0075 and DK105. In total, we identified ~46,200–48,000 consolidated gene models per line (Table 1). Cross-mapping and consolidation substantially improved the number of pairwise reciprocal best blast orthologs (Fig. 1a and Supplementary Table 6) as well as the completeness of orthologous clusters (Fig. 1b and Supplementary Fig. 2).

**Extended Data Fig. 3:**
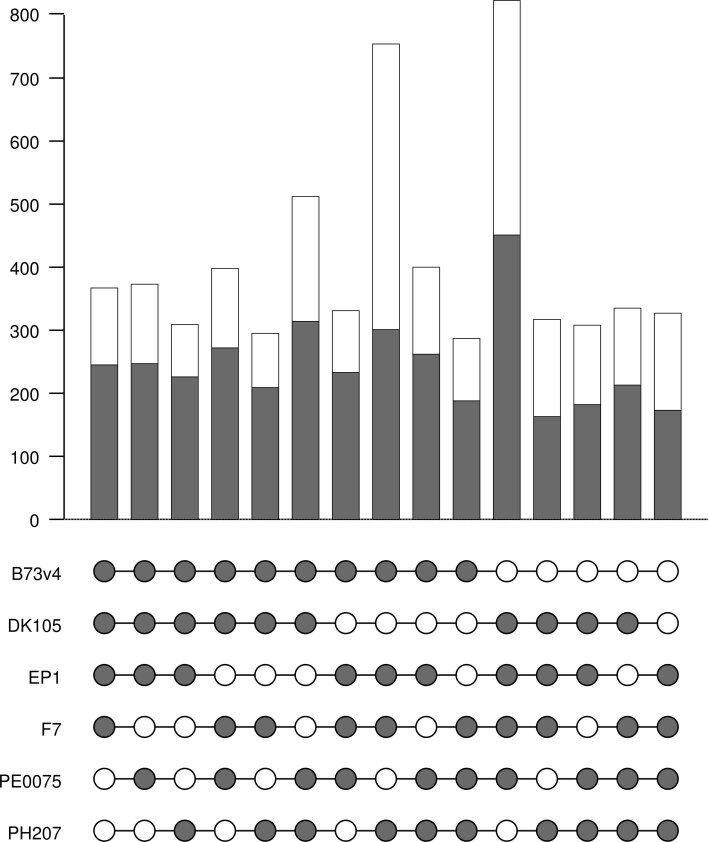
Group-specific syntelogs for all (4,2)-combinations of the six lines and their syntelog set. We divided the six maize lines of this study into two groups of 4 (gray circles) and 2 (white circles) lines, respectively. For all 15 combinations, the number of group-specific syntelogs for each group was determined under the prior of the identified orthologous gene set. The germplasm-specific separation of flint (EP1, F7, DK105 and PE0075) versus dent (B73 version 4 and PH207) genotypes showed the largest distinction between the two groups.

Overall, 43,700–45,900 genes (94.7–98% of all genes per line) have an orthologous counterpart or reciprocal best blast match in at least one of the other lines (Fig. 1b). Comparison of the original and consolidated annotations of B73 and PH207 suggests that part of this gene complement displays functional signatures such as homology to angiosperm proteins or expression in the respective line (Fig. 1a and Supplementary Fig. 3). The cross-mapped annotation significantly improved the completeness of syntelog clusters with 34,352 clusters that have syntelogs for all six lines, and 94.6% of these contain exactly one gene copy per line while 1,861 (5.4%) contain additional co-orthologous members (Fig. 1b). Genic PAVs strongly support flint and dent as distinctive germplasm (Extended Data Fig. 3). Overall, core genes present in all six maize lines show high expression levels (mean and median 25.8 and 5.9 transcripts per million, TPM) while PAV genes show significantly lower expression levels (median 0.02–0.06 TPM) and have lower homology support in a comparison to 27 angiosperms (Fig. 1c,d). Note that genes found in only one line in general show more pronounced support for protein-coding sequences compared to genes of cluster sizes 2–4. Genes with no corresponding maize ortholog (cluster size 1) are enriched for tandem duplications (Fisher exact test with *P* values between <1 × 10^−8^ and 1 × 10^−70^). The very low median expression of syntenic dispensable and singleton genes suggests no, only minor or highly specialized line-specific functions for most of these genes.

**Extended Data Fig. 4:**
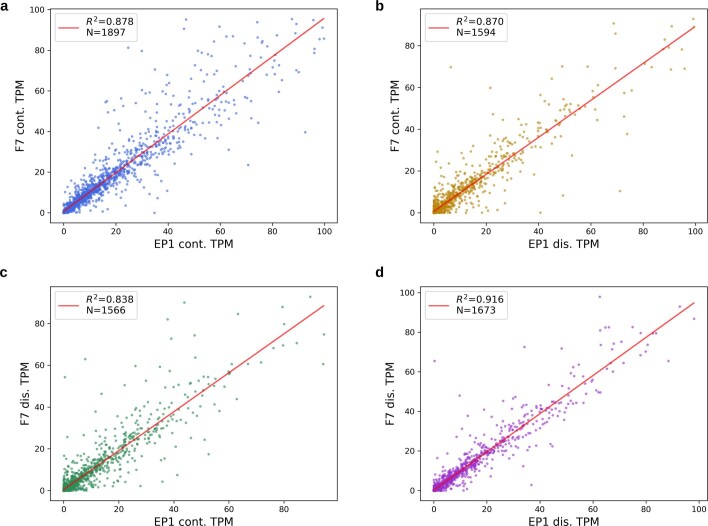
Expression levels of orthologs with contiguous and disrupted open reading frame (ORF). Consolidated mappings discovered several thousand genes for which the entire cross-mapping of the top-scoring consolidation model had no contiguous but a disrupted ORF. These genes exhibit highly similar expression levels to their nondisrupted orthologs illustrated here for lines EP1 and F7. **a**, shows the control correlation and linear regression (red line) of a random sample of F7 and EP1 orthologous pairs for which both genes have a contiguous ORF. To obtain comparable sample sizes and expression ranges, random sampling was restricted to gene pairs with a maximal TPM ≤ 100. **b**, and **c**, display expression correlations between gene pairs with exactly one disrupted and one contiguous ORF, while in subfigure **d**, both orthologous partners contain a disrupted ORF. N: number of pairs, r2: coefficient of determination.

Consistent with the genome analysis of the maize inbred line Mo17 (ref. ^17^), our cross-consolidation revealed a sizeable number of predicted genes with large effect mutations. Between 2,487 (B73) and up to 4,264 (DK105) gene models had no contiguous ORF due to internal stop codons or frameshifts. Genes with disrupted ORFs had highly similar expression levels (Pearson correlation 0.93 < *r* < 0.95) to their syntenic partner with contiguous reading frame (Extended Data Fig. 4). Close inspection revealed a series of reasons including pseudogenization, missing splice variants or skipped exons in the respective line.

#### Dynamics of the full-length retrotransposon landscape

**Extended Data Fig. 5:**
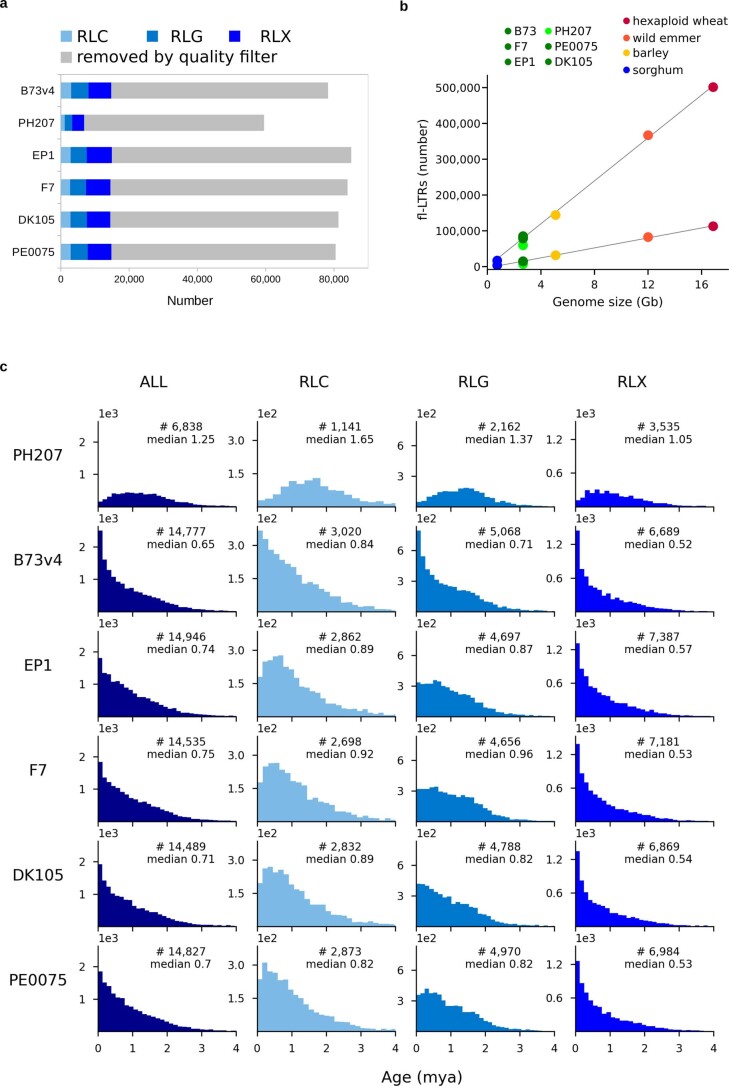
Full-length LTR-retrotransposon detection in different maize lines. **a**, *De novo* detection and stringent quality filtering of fl-LTR elements. **b**, Retrieved fl-LTR numbers as proxy for assembly quality. Upper data points give candidate fl-LTRs, lower data points give filtered, high-quality fl-LTR numbers. Five of the six assemblies contain the expected number of fl-LTRs. The by 50% reduced numbers of high-quality elements in PH207 reflect an older not optimized assembly approach. **c**, Insertion age distribution of fl-LTRs. The four NRGene assemblies have very similar age distribution patterns. The B73 assembly has a higher proportion of very young Copia and Gypsy elements, which can probably be attributed to technical assembly differences. In PH207, the young elements are completely missing in the assembly and thus explain the reduced numbers. Due to their (almost) identical long terminal repeats the correct structure of young elements is more difficult to resolve.

Approximately 80% of the maize genome is constituted by repeats of different types. In addition to the six lines above, the recently published genomes Mo17 and W22 were analyzed for their repeat content^16,17^. No pronounced differences in transposable element (TE) composition among the eight lines analyzed was detected (Extended Data Fig. 1 and Supplementary Table 7). We detected almost 15,000 high-quality full-length LTRs (fl-LTRs) per line, matching the expected genome size to fl-LTR ratio^22^ (Supplementary Table 8a). The lower number observed for PH207 (6,838 fl-LTRs) can be attributed the overall lower quality assembly compared to the other five lines. Correspondingly, the PH207 assembly was excluded from evaluation depending on the analysis criteria. For the remaining five lines, the number and age distribution of fl-LTRs confirm the high quality of the respective assemblies (Extended Data Fig. 5).

**Fig. 2:**
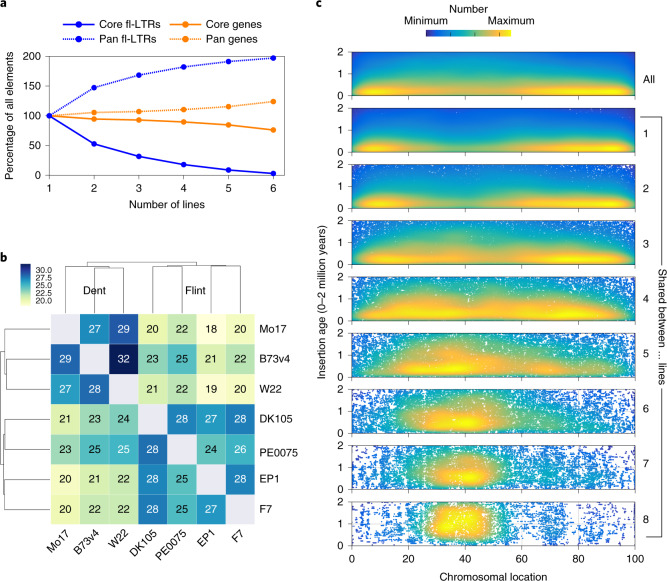
Pan and core characteristics of fl-LTR. **a**, Proportion of the pan and core sets for fl-LTRs and genes with increasing line numbers. **b**, Percentage of pairwise shared and still-intact fl-LTRs at syntenic positions for seven maize lines. Reading direction is column to row; for example, EP1 shares 27% of its fl-LTRs with F7 and F7 shares 28% with EP1. On the basis of the similarity matrix, the seven lines cluster into a relationship context that separates flint and dent. Within flint and dent, around 30% of the locations are shared; between flint and dent, most of the values are reduced to about 20%, except for PE0075 (25%). The most pronounced overlap of intact and shared fl-LTR locations is found between W22 and B73 (32%); the least pronounced is found between EP1 and Mo17 (18%). A corresponding evalution for genes gives pairwise shared numbers between 82 and 91%. **c**, Insertion age (*y* axis) and chromosomal distribution of all fl-LTRs (top row), line unique (label 1) and cluster constellations of increased sharing ranging from two to all eight lines. The chromosomal location is collapsed for all ten chromosomes and given in percentage of the respective chromosome length. The line-specific or shared among fewer lines fl-LTRs contain a higher proportion of younger elements and are less frequently found in the central, low recombining regions. There is a continuous shift towards a more pericentromeric location and towards older elements with the increase of lines sharing corresponding elements.

To quantify transposon dynamics between the different maize lines we identified still-shared syntenic fl-LTRs by clustering TE junctions with high stringency. Only 3% of all fl-LTR locations were found to be shared between six lines. While the percentage of shared elements among the different lines shows a marked decrease, the pangenome of the fl-LTR space almost doubles the number of elements accordingly. This high dynamic is in stark contrast to genes where 76.1% are retained at their syntenic position (Fig. 2a). Pairwise cross-comparison among the different genomes detected between 18 and 32% of still syntenic fl-LTRs to be present in the corresponding line. Differences in pairwise shared numbers match the phylogenies from gene-derived phylogenetic relationships and reveal a clear distinction between flint and dent lines (Fig. 2b). Line-specific fl-LTRs are younger and depleted in the pericentromeric regions. Along with increased sharing, a continuous enrichment towards the central low recombining compartments is observed and associated with an increase in age (Fig. 2c). Our findings illustrate the rapid turnover of the intergenic space most likely driven by elimination through illegitimate recombination.

#### Heterochromatic knob islands in the maize genome

**Fig. 3:**
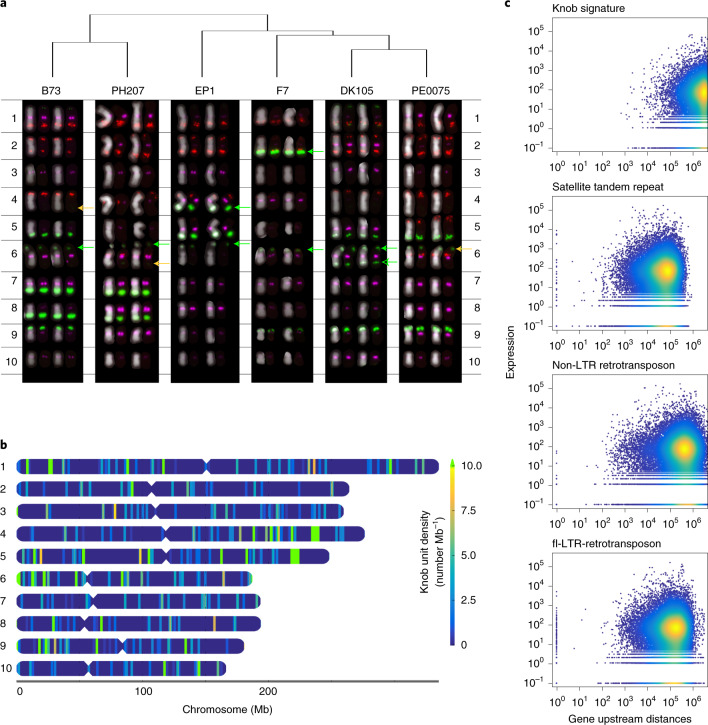
Diversity of knob locations in six maize lines. **a**, Karyotyping of all six maize lines by FISH. Colors show: red, (ACT)10; green, Knob-2 (180-bp knob repeat) and MR68-3 (chromosome 6 clone MR68, ID AF020265.1); magenta, CentC69-1. Green arrows indicate co-occurrences of Knob-2 and MR68-3; orange arrows indicate a sole occurrence of MR68-3. **b**, Chromosomal locations of all knob sequences in EP1. Besides the three major Mb scaled knob regions identified by FISH on chromosome 4L, 5L and 6S, many smaller knob sequences (<10 kb; below the FISH detection limit) are found scattered along all chromosomes. Interestingly, many of the minor knob locations are shared between the lines (Extended Data Fig. 7). The polymorphisms seen by FISH seem to be based on large size variations of the mostly syntenic minor sites (7). **c**, Relation between gene neighborhood and gene expression. For each gene the maximal expression of seven different conditions is plotted against its upstream distance (bp) to the next neighboring element of a specific type. Both axes are logarithmic. It is noteworthy that knob sequences in particular seem to be a ‘bad’ neighborhood for genes. Here, in contrast to other elements a 1,000-bp perimeter was found to be devoid of genes.

**Extended Data Fig. 6:**
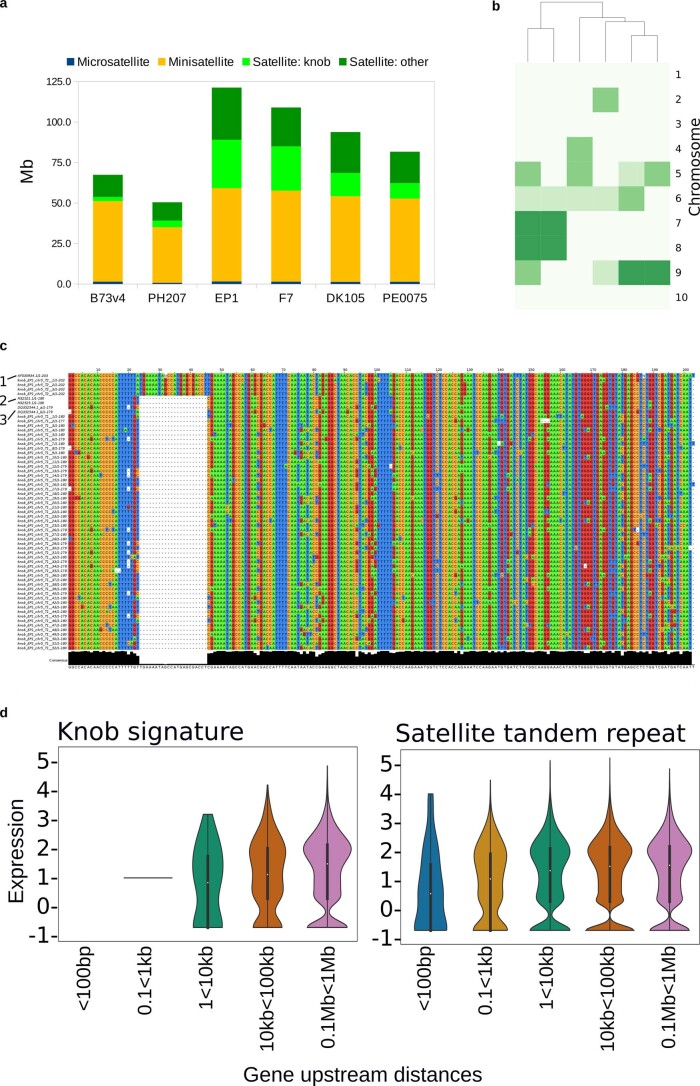
Tandem repeat content and composition in the genome assemblies. **a**, The assemblies of EP1 and F7 captured the highest amounts of tandem repeats and knob sequences. The in spite of high fish intensities much lower contents in the B73 and PH207 assemblies reflect the assembly difficulties of these highly repetitive genome regions. **b**, Relationship between the six lines derived from knob location similarities. The input matrix for clustering contained three relative intensities of fish knob locations per chromosome. The knob patterns of the two Dents B73 and PH207 are very similar, as well as the patterns of DK105 and PE0075. **c**, Multiple sequence alignment of selected knob monomers from EP1 together with known knob monomers. The knob sequences in the EP1 assembly consist of 180 and 202 bp monomers with a surplus of the 180 bp monomer by a factor of 6.8. Both monomers are highly similar to previously reported knob monomers from maize with the following Genbank IDs marked as 1, 2, 3 in the figure, 1: AF030934.1, 2: M32521.1 and M32525.1, 3: DQ352544.1_a and DQ352544.1_b. Consensus sequences of the monomers where used to identify all major and minor knob locations in the assemblies. **d**, Gene expression (maximal expression of 7 different conditions per Gene, log10) in relation to the nearest upstream knob signature (left) and tandem repeat (right). Both axes are logarithmic.

**Extended Data Fig. 7:**
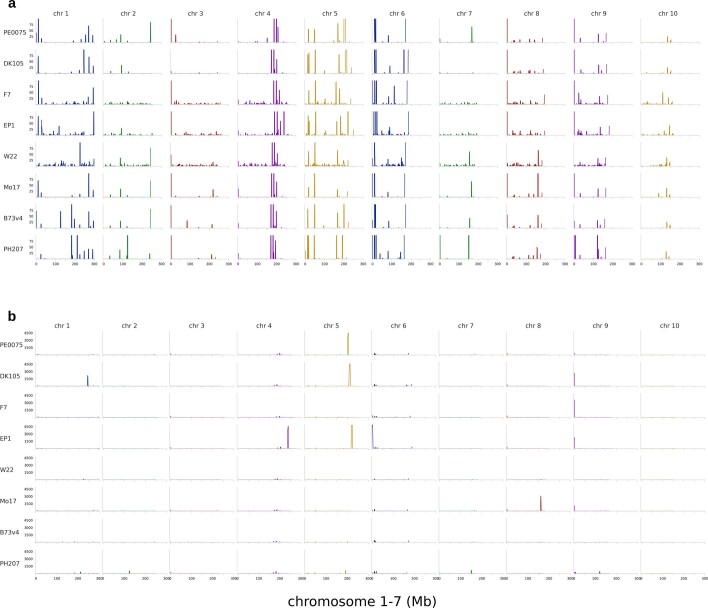
Syntenic knob signatures. x-axis: number of knob units per 2 Mb windows, y axis: chromosome length in Mb. **a**, **Minor knob locations** in the assemblies (y max=100). The majority of the minor knob locations are syntenic between the 8 lines, especially for chromosomes 4,5,and 6, and display no clear distinction between flint and dent. This is in contrast to the knob intensities and extent detected by FISH hybridizations (Fig. 3a). We hypothesize that these minor locations act as ‘anchor points’ for knob movement or expansion, which seem to occur within very short evolutionary time-spans since the separation of different breeding lines. Larger knob stretches could impact the expression of surrounding genes and influence local recombination rates. **b**, **Major knob locations** in the assemblies (Y max=4000).

Knob regions are heterochromatic regions in the genome that have been demonstrated to affect local recombination^26,27^. They belong to the group of satellite tandem repeats which are well represented in our flint assemblies (Extended Data Fig. 6a, Supplementary Fig. 4 and Supplementary Table 8b). Maize knobs are composed of 180 bp and closely related 202-bp tandemly repeated sequence units (Extended Data Fig. 6c). Variations in position and extent of knob regions in maize have been documented^26,28^. Intensity and position of knob regions detected by FISH for the different karyotypes clearly separate dent from flint lines (Fig. 3a and Extended Data Fig. 6b). In particular, extensive knob regions detected on chromosomes 7 and 8 of the dent lines are absent in flint lines. For other positions on the genome, a clear flint/dent separation among lines cannot be observed. However, the observed variations reveal the pervasive dynamics of knob regions in the genome. FISH requires a minimum number of adjacent tandem units for their detection. High-quality genome assemblies resolving a larger proportion of the repetitive space allow analyzing the so-far-hidden, cryptic, knob sites which we annotated by homology to the 180 and 202 bp monomers. Besides the large knob regions detected by FISH, numerous additional positions with fewer tandem repeat units are found in the sequence assembly on all chromosomes (Fig. 3b and Extended Data Fig. 7). A strikingly pronounced syntenic conservation of minor knob sites common to all analyzed flint and dent lines was observed. Given the observations of rapid diversification based on repeat units detailed above, this might indicate numerous potential (shadowed) knob regions that can rapidly expand or shrink by, for example, illegitimate recombination and can cause recombinational isolation of the affected regions.

In fission yeast, knob structures have been shown to implicate significant downregulation of surrounding genes^29^. To check for similar effects in maize we analyzed the distance of genes to the nearest knob signature in the assembly and related it to expression data^30^ generated for the lines B73, EP1 and F7. Even more pronounced than observed for other types of repeat elements, we find the close surrounding of knob repeat units to be devoid of genes (Fig. 3c). For genes surrounding knob regions, we observe a trend for lower expression values (Extended Data Fig. 7d).

#### Whole-genome alignment

Numerous large structural variations (SV) in maize have been reported^11,12,14^. To gain insight into the extent of SVs in the maize flint versus dent genomes we generated pairwise whole-genome alignments (WGAs) for the six lines and postprocessed the resulting alignments to single alignment blocks (SAB) that represent the highest scoring one-to-one relationships between each genome pair. To approximate overall contiguity between two genomes, SABs were further concatenated to merged alignment blocks (MABs) if they followed a strict and unambiguous order in both genome sequences (Supplementary Fig. 5). On average, ~50% of the genome sequence aligned in each of the pairwise comparisons by SAB scoring, while WGAs (MABs) associated 80–90% (1.7–2 Gb) of the genome sequences (Supplementary Tables 9 and 10). Mean sizes of SABs and MABs were ~10 kb and ~100 kb, respectively (Supplementary Fig. 6). Evaluation of pairwise WGA regions strongly supported orthology of segments in parallel orientations (Supplementary Fig. 7) while an assessment of most rearrangements (inversions and transpositions) was inconclusive due to insufficient or ambiguous information at the breakpoints.

**Fig. 4:**
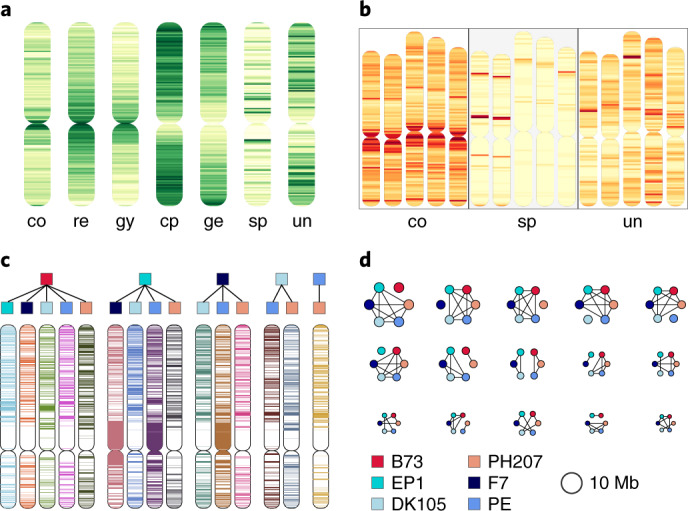
Comparative genome characteristics for the six maize lines. **a**, The density distribution of the WGA (co, core; sp, germplasm-specific; un, unaligned regions) and functional genomic elements (re, all repeats; gy and cp, gypsy and copia LTR elements; ge, genes). The core WGAs are significantly positively correlated to the repeat and gypsy densities, similar to densities of copia elements and genes. For the unaligned and germplasm-specific densities, we detected no highly significant correlations with one of the other functional elements. **b**, Densities of the core, germplasm-specific and unaligned WGA regions showed similar distributions for all maize lines, exemplified for chromosome 1 of B73, PH207, EP1, F7 and PE0075. Core regions are enriched at (peri-)centromeric regions, germplasm-specific obviously cluster in a group-specific manner and unaligned parts are random within all six lines. **c**, Runs-of-identity for SNPs from core regions for chromosome 7 and all 15 pairwise combinations. Color code above the chromosome pictograms follows legend in **d**. **d**, Sizes of higher order haplotypes of the six lines. Pictograms are proportional to their total genomic span, the legend provides the color coding of each of the six lines. Black lines indicate identity. Interestingly, the top six higher order haplotypes each group one maize variety as outlier and five lines that are identical by their SNP runs for these genomic segments. Of the more complex groupings, the most prominent haplotype separates the two germplasm, flint and dent.

**Extended Data Fig. 8:**
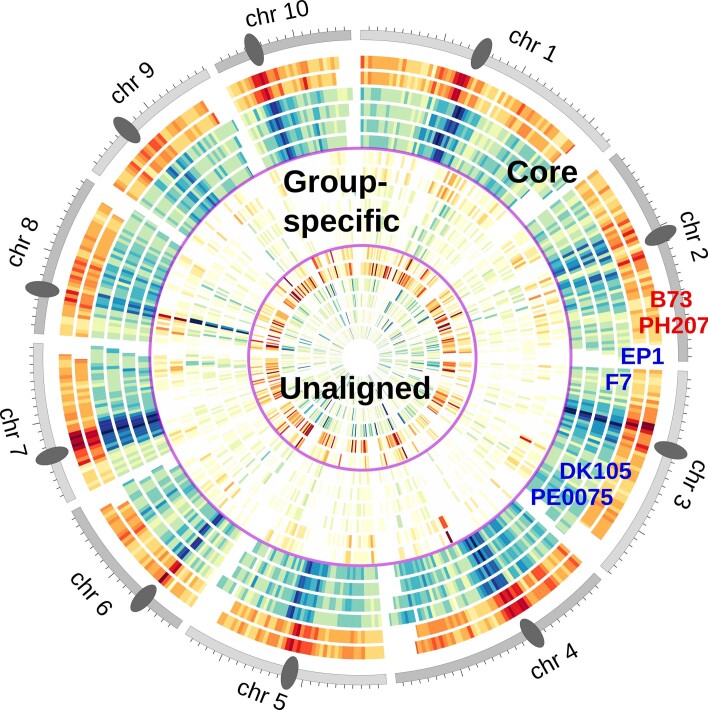
Densities of the core, group-specific and unaligned regions for the six maize lines studies. Intensities are normalized within one track, dark red/blue values representing a higher fraction of aligned regions per 5 Mb bins. Outer chromosome ideograms show the largest of all six lines, dark gray ellipses indicate the approximate centromere position derived from the repeat analysis. Group of six circular tracks show from outer to inner the core, germplasm-specific and unaligned densities. Each group consists of six tracks, displaying from outer to inner densities for B73, PH207, EP1, F7, DK105 and PE0075.

To delineate regions that do not align with any other of the five lines (unaligned), align solely with flint or dent maize lines (group-specific) or are aligned in all pairwise alignments (core), we combined pairwise WGAs of each line and projected them onto its genome sequence (Supplementary Fig. 8). We classified a genomic region as core type if it aligned to four out of the five possible lines due to the high amount of gap sequences in PH207 that likely lead to an underestimation of the true core genome. The core genome defined by SABs comprised on average ~850 Mb (40%) of the total genome while 71 Mb (3.3%) were group-specific and ~460 Mb (22%) were unaligned. This indicates a large fraction of uniquely inserted or deleted sequences in each line. Positional analysis of genomic bins revealed significant positive correlations (Kendall’s *τ* ≥ 0.38; *P* < 10^–36^) between the core and repeat density while gene densities were negatively correlated (−0.4 < *τ* < −0.33; *P* < 10^–26^) (Fig. 4a and Extended Data Fig. 8). Notably, for all six lines highest core densities located at regions adjacent to the centromere and correspond to above-average SAB sizes and below-average recombination rates (Fig. 4b). These findings are reminiscent of observations in the Triticeae gene space^22^ mirroring the recombinogenic properties of centromeric and telomeric regions.

#### Genetic mosaicism in US dents and European flints

**Extended Data Fig. 9:**
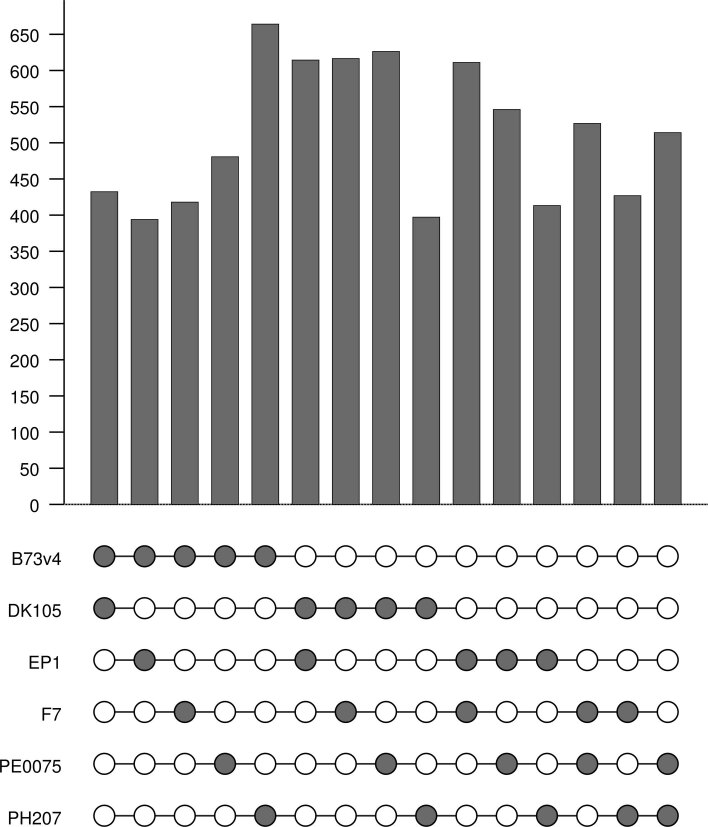
Genomic sizes in [Mb] of SNP runs-of-identity (RoI) for all 15 pairwise comparisons of the six maize lines. Comparisons within one germplasm (flint: EP1, F7, DK105 and PE0075; dent: B73 version 4 and PH207) share larger genomic fractions than inter-germplasm RoI totals. Y-axis: total sum of RoIs in [Mb], X-axis: line pairs, gray circles indicate maize line used in the respective comparison.

The core genomic regions served as positional anchors to generate multiple sequence alignments. One-to-one unambiguous alignments for the six lines total 287 Mb (~15%) of each genome and comprise 6.25 × 10^6^ orthologous SNPs with positional information for each of the six genomic coordinate systems. The detected SNPs showed an excellent agreement of 99.3% (4,827 mismatches out of 653,398 total scored calls) to a published set of SNP calls using the Affymetrix Axiom Maize Genotyping Array with 600,000 variants^31^. On the basis of sequences of identical SNP pairs, we determined significant haplotypes between all 15 line combinations (Fig. 4c). Mean and total genomic spans covered by these pairwise near-identical haplotypes range from 40 to 68 kb and 394 to 664 Mb, respectively. The proportion of one-fifth to one-third shared genomic regions between flint and dent lines is in close agreement with previous studies of NF content in stiff stalk and nonstiff stalk dents^2,6^. Number, mean and total sizes were highly consistent with their phylogenetic relationship deduced from high-confidence orthologs (Extended Data Fig. 9). However, each line of one group (flint or dent) shared substantial genomic portions with the other germplasm group (394 to 480 Mb for B73 and EP1 or PE0075, respectively). This is in line with the history of US Corn Belt dents that originated from crosses of Southern dents and flints of North America and Canada^6^. The latter established many founder lines of modern European flint breeding and were introduced to Europe as early as the discovery of the New World^8^. Alternatively, these haplotypes could be introgressions of US germplasm into European flint material that started in the 1950s to broaden genetic diversity in European breeding programs. Notably, the DH line of the European landrace Petkuser showed the largest genome-wide similarity of all flints towards B73.

Next, we surveyed higher order haplotypes considering all 15 pairwise similarities simultaneously as combined binary patterns. Limiting these haplotypes to a minimum run size of 40 SNPs, we identified 31 fitting this size criterion (Fig. 4d and Supplementary Table 11). Since the identified regions exceed random expectation by far and show near-complete SNP identity across particular line subsets/combinations, these regions are likely identical-by-descent and share a common ancestor. In total, the 31 distinct higher order haplotypes span ~288 Mb and comprise >1.1 × 10^6^ orthologous SNPs. Note that ordering by the number of SNPs as well as the genomic region covered, the top five/six haplotypes encode combinatorial groupings with five out of six lines being identical. The haplotype distinguishing between the studied dent and European flint lines ranked only as the sixth (by size) or seventh (by SNP number) most frequent (Fig. 4d and Supplementary Table 11). The distinct higher order haplotypes distribute evenly along the ten chromosomes but also comprised several striking clusters. Although not the most prominent identical-by-descent type, three regions were enriched for the haplotype distinctive between flint and dent lines: two at the distal sites of chromosomes 4 and 7 and a large portion spanning ~12 Mb in the proximal part of chromosome 8. The latter strongly coincides with group-specific WGAs on chromosome 8 (Extended Data Fig. 8) and encloses the major flowering time quantititive trait locus *vgt1* in maize^32,33^.

#### Haplotype-informed differential gene expression analysis

**Fig. 5:**
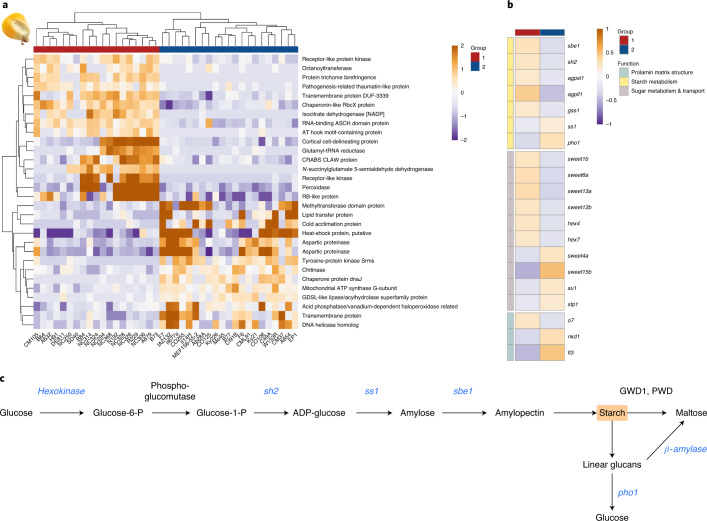
Haploblock-informed DEGs. **a**, Heatmap of top 30 DEGs (log_2_-fold change above 2 or below –2, adjusted *P* *≤* 0.05, variance stabilized) between the two haploblock-informed groups in kernels. Group 1, lines with haploblock-similarity to B73 (dent-alike); group 2, lines with haploblock-similarity to EP1/F7 (flint-alike). **b**, DEGs between the two groups that encode for components of the starch biosynthesis pathway, for proteins involved in sugar metabolism and transport into the kernel, and for proteins organizing the prolamin matrix structure of the kernel. *sbe1*, starch branching enzyme 1; *agpsl1*, ADP-glucose pyrophosphorylase small subunit leaf1; *sh2*, *shrunken2*; *ss1*, starch synthase 1; *pho1*, starch phosphorylase 1; sweet, sugar transporter; *su1*: *sugary1*; *stp1*, sugar transport protein 1; hex, hexokinase; *o7*, *opaque7*; *nkd1*, *naked endosperm*; *fl3*, *floury endosperm 3*; *PWD*, phosphoglucan, water dikinase; *GWD1*, glucan, water dikinase 1. **c**, Main reactions of starch biosynthesis and catalyzing enzymes, genes differentially expressed between the two groups of maize lines highlighted in blue.

To explore putative functional consequences of the haplotype differentiating our dent and European flint lines, we analyzed its effect on the maize transcriptome. From the maize association panel we selected a subset of 40 out of 282 lines, which were highly similar to either B73 or F7/EP1 within regions defined by this haplotype (Supplementary Fig. 9)^34,35^. We analyzed genome-wide expression levels between the two contrasting groups underlying three different genome references (B73, EP1 and F7) and using expression data from seven different conditions and tissues^30^. In total, 4,761 genes orthologous between B73, EP1 and F7 (out of 35,389 triplets) were differentially expressed (DEGs) in all three references and in at least one of the tissues and conditions (Fig. 5).

Comparing identified DEGs with curated maize genes revealed a striking pattern for genes involved in the establishment of the kernel texture. We identified a plethora of genes involved in starch metabolism: sugar transporters including several proteins of the SWEET family, *hex4* and *hex7*, sugar transporter *stp1*, the rate-limiting gene for starch biosynthesis (*shrunken2*), *starch synthase1* (*ss1*), *starch phosphorylase*, the α(1 → 6)-glucosylhydrolase *sugary*^36^ and starch branching enzyme *sbe1* catalyzing the formation of α(1 → 6)-glycosidic bonds in amylopectin (Fig. 5c). We also detected a number of genes that establish and regulate the prolamin content and structure of the maize endosperm (Fig. 5b) including *opaque endosperm o7* and *o10*, *empty pericarp 16*, *floury endosperm fl3* and *naked endosperm nkd1*^37–40^.

## Discussion

The comparative analysis of the gene and repeat content together with the analysis of WGAs and SNPs confirms the European flints as highly differentiated germplasm of cultivated maize at a whole-genome scale. For all these analyses, the four flint and the two dent lines form two distinct groups, with each line sharing larger numbers of syntenic genes, repeats, aligned regions and haplotypes with members of the same group than with any member of the other group. Nevertheless, we also observed significant overall conservation of chromosome structure and gene content. Aligned blocks ordered by consecutive syntenic order span ~85% of the entire genome in all lines. Cross-consolidation of gene models revealed a moderate number of nonsyntenic genes, suggesting that genic PAVs are less pronounced in maize than previously reported. Consistently, significant lower support by expression and homology indicates that less conserved orthologous clusters are likely enriched for nonfunctional and poor gene models. Nevertheless, the latter set also contains many genes expressed at moderate to high levels, which might contribute to line-specific adaptations and are of interest for maize improvement and breeding. Additionally, some of these genes may have highly confined expression patterns or conditions that were missed in our transcriptome analysis. The cross-consolidation approach also had several limitations including the potential transfer of poor gene models and the identification of genes that contain large effect mutations and disrupted ORFs. Inspection of the latter set revealed several causes including likely true pseudogenizations or truncations, probable differences in the exon–intron structure between the applied informant and target gene model as well as mapping errors.

In contrast to the overall syntenic conservation, a large amount of unaligned, nonorthologous sequences for WGAs and the analysis of the repeat space demonstrated enormous diversity and dynamics of the maize genome. Only 2% of shared orthologous fl-LTRs are conserved in eight lines. Consistent with previous reports, large FISH visible knobs display substantial variation in position among the maize lines studied^28,41^. Remarkably, both shared sets of orthologous LTRs and larger knob positions perfectly reproduced the relationship of the six lines based on coding sequences, indicating smooth transitions between these features during breeding and admixture. In contrast to previous studies reporting low knob numbers in NF or even knob-less NF lines, the European flints contained similar knob numbers to the US dents^41^. It is unclear whether this is a characteristic of the NF ancestral lines migrated to Europe, reflects the breeding history in European flints or represents a technical advantage given the progress in methodology to detect weaker signals. Consistent with fiber-FISH studies, we also detected numerous locations of the 180-bp knob repeat unit throughout the chromosome assemblies^42^. The potential contribution of these widespread knob repeats on gene density, expression levels and rare deleterious alleles requires future studies and adds further complexity to known effects of large-scale knobs in maize on recombination rates, meiotic drive and phenotypic traits such as flowering time^26,43,44^.

WGAs enabled us to identify long runs of pairwise and higher order combinations of genomic regions with identical haplotypes. Size and distribution of these haplotype blocks reflect complexity of historic recombination, intercrossing and breeding history over the last centuries^3,5,7,19,45–47^. Similar to the gene and repeat comparisons, the SNP runs highlight the separation of flints and dents of this study but also illustrate and delineate candidate regions of common ancestry in the US Corn Belt and European flint lines. We identified a set of regions that differentiated the studied Corn Belt dents from the European germplasm. Intriguingly, genes differentially expressed between two groups of maize varieties that have been selected by their genotypic similarity to these regions comprised a nearly complete starch biosynthesis pathway and several genes involved in the establishment and organization of the endosperm texture. Hence, besides the detection of selective sweeps and other genomic signatures, it might be equally informative to survey *trans*-effects of such genomic regions.

Given the high dynamics and tessellation of the maize genome, we estimate that many high-quality genome sequences will be required for improving breeding efficacy and understanding maize biology. The four European flint lines of this study close an important gap in our knowledge and complement the maize pangenome.

## Methods

### Plant material and genome assembly

The four flint inbred lines were chosen to represent landraces from different European ancestry. While PE0075 (a doubled-haploid line derived from the landrace Petkuser Ferdinand Rot) and DK105 (derived from Gelber Badischer Landmais) can be classified as a representatives of the NF, EP1 and F7 represent Pyrenean–Galician ancestry and were derived from populations Lizargarate and Lacaune, respectively^48^. Contigs, scaffolds and pseudochromosomes were assembled de novo using the DeNovoMAGIC 2.0 technology for lines EP1 and F7, and DeNovoMAGIC 3.0 for lines DK105 and PE0075 (Energin R Technologies 2009), respectively (Supplementary Table 2) using the B73 (v.4) reference genome to assist anchoring and orienting scaffolds and contigs to pseudochromosomes. The application of this toolset has been described previously for several plant genome assemblies^15,16,49^. Briefly, bulked leaf tissue from 15–24 seedlings was harvested, immediately ground in liquid nitrogen and genomic DNA was then isolated using a modified CTAB protocol^50^. Illumina sequencing of paired-end and mate-pair libraries was performed as specified in Supplementary Table 1. Remappings of the paired-end and mate-pair libraries were used to estimate gap sizes in the assemblies (Supplementary Fig. 10).

A cross of parental lines PH207 × EP1 was used to establish a high-density genetic map from an F_2_ mapping population. Parents and 192 F_2_ plants were analyzed using the Affymetrix Axiom Maize Genotyping Array and data were processed as described in ref. ^31^, which resulted in 174,616 markers. The markers were clustered into groups showing identical segregation patterns using POPSEQ^51^. A first genetic map was calculated with 9,404 binmap markers with the R package ASMap v.1.0-2 (ref. ^52^) using the function mstmap with the following parameters: pop.type=“RIL2”, dist.fun=“kosambi”, objective.fun=“COUNT”, p.value=1e-22, noMap.dist=15, noMap.size=2, miss.thresh=0.00. Linkage groups were assigned to the ten maize chromosomes on the basis of previously mapped markers. Unlinked small groups with only a few markers were discarded. Ten F_2_ plants exhibited very high numbers of crossovers and were excluded from further analyses. In multiple rounds of mapping using the same parameters as stated above, markers with highly distorted segregation or which led to double-crossovers were identified using the function statMark in the ASMap R package and discarded before final map construction. The final genetic linkage map contained 8,869 markers. All markers from the initial dataset which had a Hamming distance of 0 with one of the mapped binmap markers were inserted into the map, resulting in a genetic map with 174,071 markers.

### Gene annotation and consolidation

We predicted protein-coding structures for F7 and EP1 as consensus models using an approach as previously described^53,54^. Briefly, consensus gene models are based on transcriptome evidences from F7 and EP1 RNA-seq data generated within this study (see RNA preparation and Expression analysis) and protein homologies of known monocotyledonous protein sequences (maize B73 and PH207, *Sorghum bicolor* v.3.1., *Oryza sativa* v.7 MSU and *Brachypodium distachyon* v.3.1)^53,55,56^. Evidences were mapped onto the genome assembly applying GenomeThreader^57^ using a minimum alignment coverage of 50% and seed sizes 7–10 for protein and 18 for nucleic acid matches. Before mapping, transcript data were assembled using Trinity with default parameters and Bridger with *K*-mer sizes 25 and 29 bp (refs. ^58,59^). Subsequently, these sequences were combined by the evidential gene pipeline (http://arthropods.eugenes.org/EvidentialGene) to obtain the final EP1 and F7 transcriptome assemblies. Initial consensus gene models were subsequently consolidated as described in the Supplementary Notes to derive final gene sets for the four flints and two dent lines.

### FISH and karyotyping

Maize chromosomes were prepared from root meristems of 2-day-old seedlings. Roots were cut and treated with 2 mM of 8-hydroxyquinoline solution for 3.5 h. Fixation was performed overnight at room temperature in 3:1 (ethanol:acetic acid) fixative. Slides were prepared according to ref. ^60^ and in situ hybridization was performed as described in ref. ^61^. Oligonucleotide probes for karyotyping and identification of knob repeats were chosen from ref. ^62^.

Images were taken using an Olympus BX61 microscope equipped with an ORCA-ER CCD camera (Hamamatsu). All images were acquired in gray scale and pseudocoloured with Adobe Photoshop CS5 (Adobe Systems). Karyotyping was done according to ref. ^62^ using the maize line B73 as a reference.

### Repeat analysis

To obtain equivalent transposon and tandem repeat data for comparative analyses we annotated all six lines with the same annotation workflows. An homology search against the Panicoidae section of the PGSB transposon library^63^ resulted in a basal transposon annotation. The REdat_9.8_Panicoideae used contains publicly available Panicoideae transposons templates as well as de novo detected full-length LTR-retrotranspons from maize (12,510 elements from B73 (v.2)) and sorghum (3,368 elements). The program vmatch (http://www.vmatch.de) was used as a fast and efficient matching tool well suited for large and highly repetitive genomes under the following parameter setup: identity ≥70%, minimal hit length 75 bp, seedlength 12 bp (exact commandline: -d -p -l 75 -identity 70 -seedlength 12 -exdrop 5). The vmatch output was filtered for redundant hits via a priority-based approach, which assigns higher scoring matches first and either shortens (<90% coverage and ≥50-bp rest length) or removes lower scoring overlaps. The resulting annotation is free of overlaps. Elements that have been interrupted by other transposon insertions (nesting) are not defragmented into a higher order instance (such as exons belonging to one gene).

Full-length LTR-retrotransposons (fl-LTR) where identified with LTRharvest^64^ using the following parameters: overlaps best -seed 30 -minlenltr 100 -maxlenltr 2000 -mindistltr 3000 -maxdistltr 25000 -similar 85 -mintsd 4 -maxtsd 20 -motif tgca -motifmis 1 -vic 60 -xdrop 5 -mat 2 -mis -2 -ins -3 -del -3. All candidates from the LTRharvest output were subsequently annotated with PfamA domains using hmmer3 (http://hmmer.org) and stringently filtered for false positives by several criteria, the main ones being the presence of at least one typical retrotransposon domain (for example, reverse transcriptase (RT), RNase H (RH), integrase (INT), protease (PR) and so on) and a tandem repeat content <25%. The inner domain order served as a criterion for the classification into the Gypsy (RT–RH–INT) or Copia (INT–RT–RH) superfamily abbreviated as RLG and RLC. Elements missing either INT or RT were classified as RLX. The insertion age of each full-length LTR-retrotransposon was estimated on the basis of the accumulated divergence between its 5′ and 3′ LTRs and a random mutation rate of 1.3 × 10^–8^ (ref. ^65^).

Tandem repeats were identified with the TandemRepeatFinder under default parameters^66^ and subjected to an overlap removal as described above, prioritizing longer and higher scoring elements. *K*-mer frequencies were calculated with Tallymer^67^.

Syntentic fl-LTRs where identified by sequence clustering (vmatch dbcluster, 98% identity and 98% coverage) of TE junctions from the de novo annotated fl-LTR locations from each line. The junctions consisted of 2 × 100-bp sequence signatures spanning the upstream and downstream insertion sites with each 50 bp inside and 50 bp outside of the TE element. The LTR assembly index was calculated using the LTR_retriever program suite^68^ as suggested on the Github pages of LTR_retriever (github.com/oushujun/LTR_retriever). LTR_FINDER (ref. ^69^) was run via the wrapper LTR_FINDER_parallel^70^ using the following parameters: -w 2 -C -D 15000 -d 1000 -L 7000 -l 100 -p 20 -M 0.85 (20 threads and 5-Mb batches). LTRHarvest^64^ was run with: -minlenltr 100 -maxlenltr 7000 -mintsd 4 -maxtsd 6 -motif TGCA -motifmis 1 -similar 85 -vic 10 -seed 20.

### Whole-genome alignments

Block alignments of high identity were generated for all pairwise combinations of the six maize lines using the MUMMER v.3 suite^71^. Initial alignments were computed using nucmer with a minimal cluster size of 250 bp and a seed size of 20 bp. Results were piped through the delta-filter tool selecting the best one-to-one blocks (global option -1) with a minimal size of 500 bp (-l 500). Despite this filter step, many (~41,000–81,000) putatively translocated blocks aligned small regions between different chromosomes or unanchored scaffolds. These blocks frequently overlapped ‘regular’ blocks linking same orthologous chromosomes and showed significantly lower sequence identities in comparison to their respective regular blocks with which they overlapped. Hence, they likely represented paralogous alignments triggered by tandem repeats or the absence of the truly orthologous sequences in one of the lines. To derive the final set of SAB, we only included candidate interchromosomal translocations with a minimal sequence identity of 99% and a maximum overlap of 10 bp to its adjacent alignments. To gain an overview of the contiguity between the six maize genomes, SABs were connected to MABs if SABs were directly adjacent in both genomes with a consistent orientation (Supplementary Fig. 11).

To identify and delineate genomic regions for each line that aligned (1) to none of the other lines (designated as ‘unaligned’), (2) to all lines of the same group (either flint or dent maize) and to none of the other group (aka ‘group-specific) or (3) to all five other lines (‘core6’), we superimposed SABs and MABs of one line on its genome coordinates by recording from which group and how many times a single genomic base participated in an alignment. Thereby, we classified each base and concatenated adjacent bases of the same type (1–3) to derive the unaligned, group-specific and core genomic part. Reversing this approach, identified core regions of EP1 were reprojected onto the pairwise alignments to decode the sequence and coordinates of matching core elements in the other five lines. On the basis of these coordinates, sequences for each core block were extracted and aligned applying the Fast Sequence Aligner^72^ with default parameters.

### Determination of paired and higher order haplotypes

SNPs were directly determined from multiple sequence alignments of the WGA core blocks (omitting insertions/deletions). Their position in each of the six genomes was derived from the position in the alignment and the known genomic start- and end-positions of the respective core block. Given the observed SNP frequencies, and applying randomization studies and run-of-head statistics, the expected maximal run lengths of identical SNPs between two lines range from 21 to 30 consecutive SNPs. We selected genomic windows of ≥40 identical SNPs as seeds to identify shared haplotypes of likely common ancestry and used those as seeds for a greedy extension to delineate genomic segments with ≥98% sequence identity between all 15 pairwise line combinations. To deduce higher order haplotypes, we transformed the 15 pairwise runs-of-identities to binary SNP patterns and surveyed seeds with ≥40 consecutive identical binary configurations. An iterative greedy algorithm linking the seed with adjacent upstream and downstream runs extended such seeds if the run exhibited a haplotype identical to the seed and less than three nonmatching SNPs were observed between the current end of the extension and the candidate run.

### RNA preparation

Different tissues of the European maize (*Z. mays*) inbred lines EP1 and F7 were sampled according to refs. ^73,74^ and subsequently subjected to transcriptome sequencing (Supplementary Table 5). Samples 1–3 were collected from seeds imbibed (whole seed, sample 1) or germinated (primary root, sample 2; coleoptile, sample 3) in paper rolls^75^ in a 16 h light (28 °C) and 8 h dark (21 °C) regime of a growth cabinet (Conviron CMP6010, http://www.conviron.com). Tissue samples 4–24 were taken from plants grown in a climate chamber either in small pots (14 cm top diameter, 8 cm height, 0.25 l volume for samples 4–17) or in big pots (28 cm top diameter, 21 cm height, 10 l volume for samples 18–24) containing soil substrate type ED 73 (https://www.meyer-shop.com). Growth conditions in the climate chamber were 16 h light (28 °C) and 8 h dark (21 °C). Two individual plants were collected for each of the 24 tissues. Harvested plant material was immediately frozen in liquid nitrogen and stored at −80 °C until RNA extraction. For total RNA extraction, the 24 samples per genotype were separately ground in liquid nitrogen. Subsequently, one spatula of each of the 24 samples per genotype was mixed to generate one pool per inbred line EP1 and F7, respectively. Total RNA was extracted from each of the two pools using the Qiagen RNeasy mini kit according to the manufacturer’s protocol (Qiagen, https://www.qiagen.com), including on-column DNA digestion. RNA quality was determined by agarose gel electrophoresis and by a Bioanalyzer using an Agilent RNA 6000 Nano Chip (Agilent Technologies, https://www.agilent.com). Both RNA samples were of excellent quality with RNA integrity number values^76^ between 9.8 and 10. In total, 9 µg of total RNA per pool were used for RNA-seq at Novogene (Novogene). After mRNA enrichment using oligo(dT) beads, sequencing libraries were constructed with the NEBNext Ultra RNA Library Prep Kit for Illumina (New England Biolabs) and Illumina PE (150 bp × 2) sequencing was performed on a HiSeq4000 by Novogene. The number of raw reads and quality-filtered reads are shown in Supplementary Table 12. Reads containing adapters, reads containing >10% undefined bases and reads containing >50% low quality bases (Qscore 5) were removed.

### Expression analysis and analysis of DEGs

Genomic variants overlapping the flint/dent specific haplotype identified in this study were extracted from the Maize Hapmap v.3.2.1 panel^34^ for lines of the 282 inbred maize association panel^77^. Selected variants were subjected to phylogenetic analysis using FastTree (v.2.1.5 SSE3)^78^ with default parameters and the resulting phylogenetic tree was visualized using iTOL^79^ (Supplementary Fig. 18). Two nonoverlapping subtrees were selected such that three of the lines presented in this study (B73, EP1 and F7) are contained, together with other topologically close lines. All lines in the subtree containing B73 were assigned to group one; all those in the subtree containing both EP1 and F7 were assigned to group two.

For the resulting subset of 41 lines (Supplementary Fig. 9) samples from seven different tissues (germinating shoot and root, the tip and base of leaves as well as leaf samples under light and dark conditions and kernels) were downloaded from SRA (PRJNA383416)^30^. Read preprocessing was performed as described in the Lexogen QuantSeq user guide (https://www.lexogen.com/wp-content/uploads/2018/10/015UG108V0201-QuantSeq-Data-Analysis-Pipeline.pdf). Trimmed reads were mapped to the maize reference sequences B73 (v.4), EP1 and F7 using STAR aligner v.2.5.1a (ref. ^80^) applying parameters outFilterMultimapNmax=10, outFilterMismatchNoverLmax=0.04,outFilterIntronMotifs=RemoveNoncanonicalUnannotated To obtain nonunique gene-level counts from the mapping files, HTSeq (v.0.11.2) with the ‘nonunique all’-method was used^81^. Normalization of read counts was performed by library sequence depth using the R package DESeq2 (v.1.23.3)^82^. For differential gene expression analysis with DESeq2, lines classified into either the flint- or dent-like group were treated as biological replicates. In this study, we report only genes for which all three orthologs of RNA-seq read mappings to B73, EP1 and F7 were significant at Benjamini–Hochberg adjusted *P* ≤ 0.05.

### Reporting Summary

Further information on research design is available in the Nature Research Reporting Summary linked to this article.

## Online content

Any methods, additional references, Nature Research reporting summaries, source data, extended data, supplementary information, acknowledgements, peer review information; details of author contributions and competing interests; and statements of data and code availability are available at 10.1038/s41588-020-0671-9.

## Supplementary information

Supplementary Information Supplementary Notes, Tables 1–12 and Figs. 1–11

Reporting Summary

## Data Availability

The de novo assembled genomes and raw reads were released in collaboration with the Maize Genetics and Genomics Database MaizeGDB^18^ and NCBI BioProjects PRJNA360923 (F7, DK105 and PE0075) and PRJNA360920 (EP1). Additionally, RNA-seq raw data were also deposited under these NCBI accessions.
