# Supplementary material for: European maize genomes highlight intraspecies variation in repeat and gene content
Source: Nat Genet. 2020 Jul 27;52(9):950–7. doi: 10.1038/s41588-020-0671-9 (PMC7467862; doi:10.1038/s41588-020-0671-9)

nature genetics

SUPPLEMENTARY INFORMATION

https://doi.org/10.1038/s41588-020-0671-9

In the format provided by the authors and unedited.

European maize genomes highlight intraspecies variation in repeat and gene content

Georg Haberer[1], Nadia Kamal[1], Eva Bauer[2], Heidrun Gundlach[1], Iris Fischer[1], Michael A. Seidel[1], Manuel Spannagl[1], Caroline Marcon[3], Alevtina Ruban[4,5], Claude Urbany[5], Adnane Nemri[5], Frank Hochholdinger[3], Milena Ouzunova[5], Andreas Houben[4], Chris-Carolin Schön[2] ✉ and Klaus F. X. Mayer[1,6] ✉

[1]Plant Genome and Systems Biology, Helmholtz Center Munich, Neuherberg, Germany. [2]Plant Breeding, School of Life Sciences, Technical University Munich, Munich, Germany. [3]Crop Functional Genomics, Institute for Crop Science and Resource Conservation, University of Bonn, Bonn, Germany. [4]Leibniz Institute of Plant Genetics and Crop Plant Research, Seeland, Germany. [5]KWS SAAT SE, Einbeck, Germany. [6]School of Life Sciences, Technical University Munich, Munich, Germany. ✉e-mail: chris.schoen@tum.de; k.mayer@helmholtz-muenchen.de

Supplementary Information

Supplementary Notes

Annotation consolidation

Next to true biological variation, genic differences among closely related species or populations may also be due to distinct gene prediction toolchains and the depth and types of evidences applied. To minimize potential technical artifacts we employed a comparative cross-mapping strategy that complemented a considerable number of previously missed genes in all lines. Initial evidence-based gene sets (see Methods) for EP1 and F7 comprised 39,352 and 41,387 protein coding genes, respectively. Additional candidate loci for EP1 and F7, and the original representative gene models of B73 and PH207[1] were searched in all six genome sequences (including PE0075 and DK105) by blat alignments[2]. We denote gene models used for the mapping as 'informants', and those derived from informant mappings to another genome sequence as 'target' models. Homologous loci were further refined by exonerate surveys[3] spanning the genomic region detected by blat and 20 kb up- and downstream sequence. Blat and exonerate alignments showed a linear correlation allowing direct conversion and comparison of both scoring schemes (r>0.999). For each blat/exonerate target pair, the top scoring model was selected and added to the initial models if the target (i) did not overlap with existing gene models, (ii) had a contiguous ORF, and (iii) ≥95% of the informant sequence was covered by the target. The resulting gene set constituted gene set 1 (GS1). After filtering for transposon or transposon-derived genes this gene set (GS1) comprised ~45-47.5k protein coding gene calls per line.

Current gene annotations largely rely on automatic pipelines and still contain poor gene models like partial, merged or transposon-derived structures. Cross mapping bears the danger to transfer such models between each genome and thereby potentially enriches poor annotations. To estimate this effect and to evaluate gene-based statements of this study, we trained a multi-layer perceptron (MLP) classifying genes into low (LC) and high confidence (HC) models based on their consistency to known protein structures of other annotations. We applied the implementation of scikit-learn of an MLPclassifier using backpropagation learning with the stochastic gradient-based optimizer, and two hidden layers of 6 and 3 neurons, respectively[4]. A training set was based on swissprot maize proteins and a set COG clusters[5] that were constructed from bi-directional best blast hits (bbhs) between 10 plant species: *Arabidopsis thaliana, Oryza sativa, Hordeum vulgare, Triticum aestivum, Sorghum bicolor, Sertaria italica, Brachypodium distachyon, Ananas comosus, Leersia oryzoides, Phyllostachus edulis*. At least three mutual bbhs with an alignment coverage ≥95% defined a COG. The high confidence (HC) training set ('positives') consisted of maize swissprot proteins entirely matching a maize fl-cDNA[6] or B73 proteins with an alignment coverage ≥95% to one of the COG clusters above. Maize genes with matches <50% coverage to any of the COG cluster genes formed the low confidence (LC) set ('negatives'). For each gene in both sets, we constructed an 18-tuple feature vector recording identity, query and hit coverage of its top blastp match against Arabidopsis, Rice, Sorghum, Brachypodium, emmer and all curated swissprot proteins. Positives and negatives were well separated by PCA, and 10x cross-validation showed a high accuracy >99% of the MLPclassifier.

A set of 3,917 transposon genes identified from GS1 based on Interpro domains and description lines (https://github.com/groupschoof/AHRD) was excluded from training data and separately classified afterwards. The eligibility of our classifier to identify problematic gene models is demonstrated by significantly lower expression levels of LC genes (median tpm_{LC} = 0.07 versus tpm_{HC} = 6.39), lower exon number per gene (2.9 exons per LC gene versus 5.3 exons per HC gene) and a higher fraction of single exon genes (29.1% versus 21.7%; Fisher test $p < 1^{-300}$). In addition, out of 3,917 initial genes containing InterPro signatures indicative for transposons, 93% were classified as LC suggesting that the classifier is well capable to identify transposon-derived genes. It should be emphasized that LC genes cannot solely considered as biologically non-functional genes but comprise both, true genes displaying erroneous structures caused by problematic underlying evidences, sequences, gene calls etc., as well as potential over-predictions.

To derive gene set 2 (GS2), we first selected HC singletons and genes from syntelog clusters (see below) that comprised only syntelogs of maximal 5 lines and/or syntelogs differing from the CDS mean size of the respective cluster by > 5%. Each of these gene models were cross-mapped to the other five lines using the blat/exonerate alignment procedure described above. We collected all matches between the target and the informant genome that were located at a neighboring (±5 Mb) syntenic position as defined by the WGA (see below). If the informant model was located outside of an alignment, we approximated its syntenic position using WGAs framing the informant and set the midpoint in the target genome proportional to the distances to both flanking alignments. Subsequently, for each syntelog cluster we determined the top scoring combination of matches over all lines to infer gene models that optimize the fit and score for all participating lines and matches of this cluster. First, GS1 models and all selected matches were clustered into groups with congruent CDS sizes (maximal difference ±5%). For each group, we selected the top scoring model per line and summed their scores to obtain a group score. The second annotation step was then based on models of the top scoring group and replaced or complemented by the GS1 model in one or more lines of the syntelog cluster. This gene set was further cleaned for transposon derived genes by the removal of singleton genes and syntelog clusters for which more than half of the cluster genes showed Interpro domains matching transposon domains (GS2). A third round padded GS2 for (orthologous) matches that have been excluded or missed due to previously applied filter steps, for example overlaps of transcript regions with LC coding regions.

Variability in gene numbers mainly reflects underlying variations in assembly quality of the different lines. For example, the lower gene count found in PH207 is likely attributable to comparably larger gaps in the genome assembly whereas the gene count in EP1 and F7 is impaired by a higher amount of genes located in unanchored scaffolds that presumably represent assembly duplications.

To address potential limitations of our cross-consolidation and potential erroneous gene models by mapping artefacts, we classified the final gene models into confidence classes using a machine learning approach described above and further classified LC genes into three subcategories based on their homology to 27 angiosperm proteomes. Transcriptome evidence independently supported this classification (Supplementary Figure 11). Classification criteria and tags for each gene model are provided within the GFF files (see https://www.maizegdb.org).

Synteny

To determine syntelogs during the consolidation steps, we applied i-adhore v3[7] running the hybrid cluster mode, a tandem gene distance of 10 and a minimum of 5 anchors to generate higher order syntenic relations for all six maize lines. Input pairwise gene similarities were derived from all-against-all blastn searches against coding sequences (CDS). To address putative ambiguities in the syntelog assignment caused by tandem arrays and the WGD in maize, we constructed a syntelog graph G = (V,E) with gene IDs as vertices V and multiplicon pairs provided by i-adhore as edges E. Edge weights were proportional to the size of a multiplicon, ie. the number of syntelogs within one syntenic block. In case of multiple edges from one vertex/gene, we only kept the highest scoring edge(s). This rule was applied in two consecutive steps, first to intra- and then inter-line connectors. In a second post-processing step, remaining clusters with vertices having two or more links to one line were further split into maximal cliques and a score was assigned to each clique as the sum of its edge/multiplicon weights. Next, we disjoint the cluster graph into non-overlapping clique subgraphs with a top-down approach thereby keeping cliques with lower scores only if none of its nodes is contained in the already selected subgraphs. The underlying rationale to select for syntelog cliques is analogous to the bidirectional best blast hit and the COG schemes.

Final orthologous genes were computed as reciprocal or bidirectional best hits (BBH) of the pairwise blastn comparisons of predicted transcripts including disrupted genes. Orthologous clusters were defined as connected components of an undirected graph with genes as nodes and BBH relations as edges similar to the approach described above. For reporting final orthologous numbers, we also counted orthologous clusters with more than one gene per line thereby including co-orthologs.

1. Portwood, J.L., 2nd, et al., *MaizeGDB 2018: the maize multi-genome genetics and genomics database.* Nucleic Acids Res, 2019. **47**(D1): p. D1146-D1154.
2. Kent, W.J., *BLAT--the BLAST-like alignment tool.* Genome Res, 2002. **12**(4): p. 656-64.
3. Slater, G.S. and E. Birney, *Automated generation of heuristics for biological sequence comparison.* BMC Bioinformatics, 2005. **6**: p. 31.
4. Pedregosa, F., et al., *Scikit-learn: Machine Learning in Python.* Journal of Machine Learning Research, 2011. **12**: p. 2825-2830.
5. Tatusov, R.L., et al., *The COG database: a tool for genome-scale analysis of protein functions and evolution.* Nucleic Acids Res, 2000. **28**(1): p. 33-6.
6. Soderlund, C., et al., *Sequencing, mapping, and analysis of 27,455 maize full-length cDNAs.* PLoS Genet, 2009. **5**(11): p. e1000740.
7. Proost, S., et al., *i-ADHoRe 3.0--fast and sensitive detection of genomic homology in extremely large data sets.* Nucleic Acids Res, 2012. **40**(2): p. e11.

Supplementary Tables

Supplementary Table 1. Read sequences for the assemblies. Table lists library types, insert sizes, read types, number of libraries and raw read coverage for lines EP1, F7, DK105 and PE0075 used for de novo genome assembly.

Library type	Insert size	Reads	No. libraries	Coverage EP1	Coverage F7	Coverage DK105	Coverage PE0075
PCR-free PE library (PE250X2)	450-470 bp	250 bp x 2	1	109x	75x	68x	68x
PCR-free PE library (PE150X2)	700-800 bp	150 bp x 2	1	84x	44x	39x	39x
MP (Nextera™ MP Gel Plus)	2-4 kbp	150 bp x 2	1	34x	34x	37x	41x
MP (Nextera™ MP Gel Plus)	5-7 kbp	150 bp x 2	1	34x	43x	38x	33x
MP (Nextera™ MP Gel Plus)	8-10 kbp	150 bp x 2	1	59x	29x	32x	36x
**Total coverage**				**320x**	**225x**	**214x**	**217x**

Supplementary Table 2. Assembly and BUSCO statistics for the four *de novo* assemblies of EP1, F7, PE0075 and DK105. BUSCO results refer to the *Liliopsidae* dataset. Percentage of gaps is provided as total length of undefined sequences ('N') relative to the total assembly size. Gap size estimation is supported by mate-pair analysis in Supplement Figure 10.

	EP1	F7	PE0075	DK105
**DeNovoMAGIC**	*v2*	*v2*	*v3.0*	*v3.0*
**Scaffolds stats**				
Total scaffolds [#]	71,196	77,899	1,288	1,393
Assembly size [bp]	2,462,913,883	2,404,712,832	2,198,402,809	2,288,116,732
Gap count	35,558	35,853	29,255	29,339
Gaps [%]	1.03	1.06	0.66	0.62
N50 [bp]	6,134,294	9,483,449	8,642,309	10,390,014
MAX [bp]	29,676,303	43,780,026	42,352,866	40,445,661
**Contigs stats**				
Total contigs [#]	137,249	130,426	116,681	51,270
Assembly size [bp]	2,434,778,235	2,377,026,979	2,256,590,598	2,242,258,170
N50 [bp]	82,295	96,432	109,087	101,213
MAX [bp]	766,959	704,566	1,314,119	1,173,809
**BUSCO**				
Complete	3140 (95.8%)	3121 (95.2%)	3139 (95.8%)	3135 (95.7%)
Complete Single-copy	2761 (84.2%)	2,718 (82.9%)	2745 (83.8%)	2743 (83.7%)
Complete Duplicated	379 (11.6%)	403 (12.3%)	393 (12.0%)	392 (12.0%)
Fragmented	59 (1.8%)	68 (2.07%)	57 (1.7%)	65 (2.0%)
Missing	79 (2.4%)	89 (2.72%)	82 (2.5%)	78 (2.3%)
**Total searched**	**3,278**	**3,278**	**3,278**	**3,278**

Supplementary Table 3. Sequence accuracy for EP1 and F7 assemblies. Illumina reads of 30 maize lines (NCBI Bioproject PRJNA260788) were mapped by BWA to the respective reference genomes and SNPs were called using bcftools with no filtering thereby reporting all possibly variant sites. To avoid mapping artifacts for the evaluation of the EP1 and F7 sequence accuracy, we scored only sites with a read depth ≥ 10, mapping quality ≥ 20, genotype quality GQ ≥ 10 and no strand bias (SBphred == 0). Columns show per chromosome numbers of total scored sites ('sites'), and calls that show no ('consistent'), a homozygous ('homoalt') or heterozygous ('hetalt') difference to the reference sequence, respectively.

	EP1				F7			
**scaffold**	**sites**	**consistent**	**homoalt**	**hetalt**	**sites**	**consistent**	**homoalt**	**hetalt**
chr_1	7589219	7589158	8	53	8129700	8129664	9	27
chr_2	6037276	6037246	4	26	6309689	6309658	6	25
chr_3	5920655	5920620	1	34	6256886	6256850	4	32
chr_4	6671784	6671707	17	60	6853201	6853156	13	32
chr_5	5396328	5396296	3	29	5602100	5602074	2	24
chr_6	4002220	4002194	2	24	4209873	4209853	1	19
chr_7	4502468	4502434	12	22	4778508	4778484	1	23
chr_8	4427095	4427072	1	22	4653156	4653133	2	21
chr_9	4144162	4144116	4	42	4287042	4287010	11	21
chr_10	3825773	3825746	1	26	3942482	3942455	1	26
**ALL**	**52516980**	**52516589**	**53**	**338**	**55022637**	**55022337**	**50**	**250**

Supplementary Table 4. LTR assembly index (LAI) of eight maize lines. The LAI was calculated for chromosome 1 of each line with LTR_retriever using default parameters. The input candidates have been identified beforehand by LTR_FINDER and LTRHarvest under the parameter settings suggested by LTR_retriever and desrcibed in the methods section.

	raw_LAI = intact LTRs % / total LTRs %		LAI = raw_LAI + 2.18 * (94 – mean_LTR_identity)		
**line**	**LAI intact % of assembly**	**LAI total % of assembly**	**raw LAI**	**LAI**	**mean LTR identity ***
PE0075	12.05	76.41	15.8	15.0	94.28
DK105	10.61	75.68	14.0	13.0	94.38
F7	10.95	76.66	14.3	13.1	94.43
EP1	8.66	77.26	11.2	9.1	94.76
B73v4	17.09	76.12	22.5	23.3	93.71
Mo17	16.72	75.8	22.1	22.5	93.83
W22	12.55	74.94	16.8	15.7	94.37
PH207	2.1	55.38	3.8	4.2	93.87

* mean LTR identity determined by LTR_retriever via an all vs all blast of all LTR elements annotated by RepeatMasker

Supplementary Table 5. Overview of sampling for RNAseq with developmental time points, tissues/organs, number of plants sampled, growth conditions and sampling dates. DAS: days after sowing, DAP: days after pollination. Growth conditions: A – paper rolls, B - soil, small pots, C - soil, big pots.

Sample no.	Dev. time point	Tissue/organ	No. of plants	Growth cond.*
**1**	24 DAS	germinating whole seed	2	A
**2**	4 DAS	primary root	2	A
**3**	4 DAS	coleoptile	2	A
**4**	9 DAS	seminal & lateral roots	2	B
**5**	9 DAS	primary & lateral roots	2	B
**6**	9 DAS	pooled leaves	2	B
**7**	5-leaf-stage	shoot tip	2	B
**8**	5-leaf-stage	topmost leaf	2	B
**9**	5-leaf-stage	base of 4th leaf	2	B
**10**	5-leaf-stage	tip of 4th leaf	2	B
**11**	3-leaf-stage	stem and SAM	2	B
**12**	3-leaf-stage	first leaf and sheath	2	B
**13**	3-leaf-stage	topmost leaf	2	B
**14**	3-leaf-stage	all roots (primary, lateral, seminal roots)	2	B
**15**	5-leaf-stage	crown roots	2	B
**16**	5-leaf-stage	seminal & lateral roots	2	B
**17**	5-leaf-stage	primary & lateral roots	2	B
**18**	1 day before pollination (R1)	non-flowering tassel	2	C
**19**	1 day before pollination (R1)	silk	2	C
**20**	1 day before pollination (R1)	innermost husk	2	C
**21**	1 day before pollination (R1)	uppermost leaf	2	C
**22**	1 day before pollination (R1)	shoot-borne roots	2	C
**23**	1 day before pollination (R1)	pre-pollination comb	2	C
**24**	4 DAP	whole seed	2	C

Supplementary Table 6. Pairwise reciprocal best blast hit orthologs for consolidated and original gene annotations. Pairwise match counts are provided in 103, B73ori, PH207ori and B73con and PH207con represent public annotations version v4 and v1.1 and the consolidated annotations, respectively.

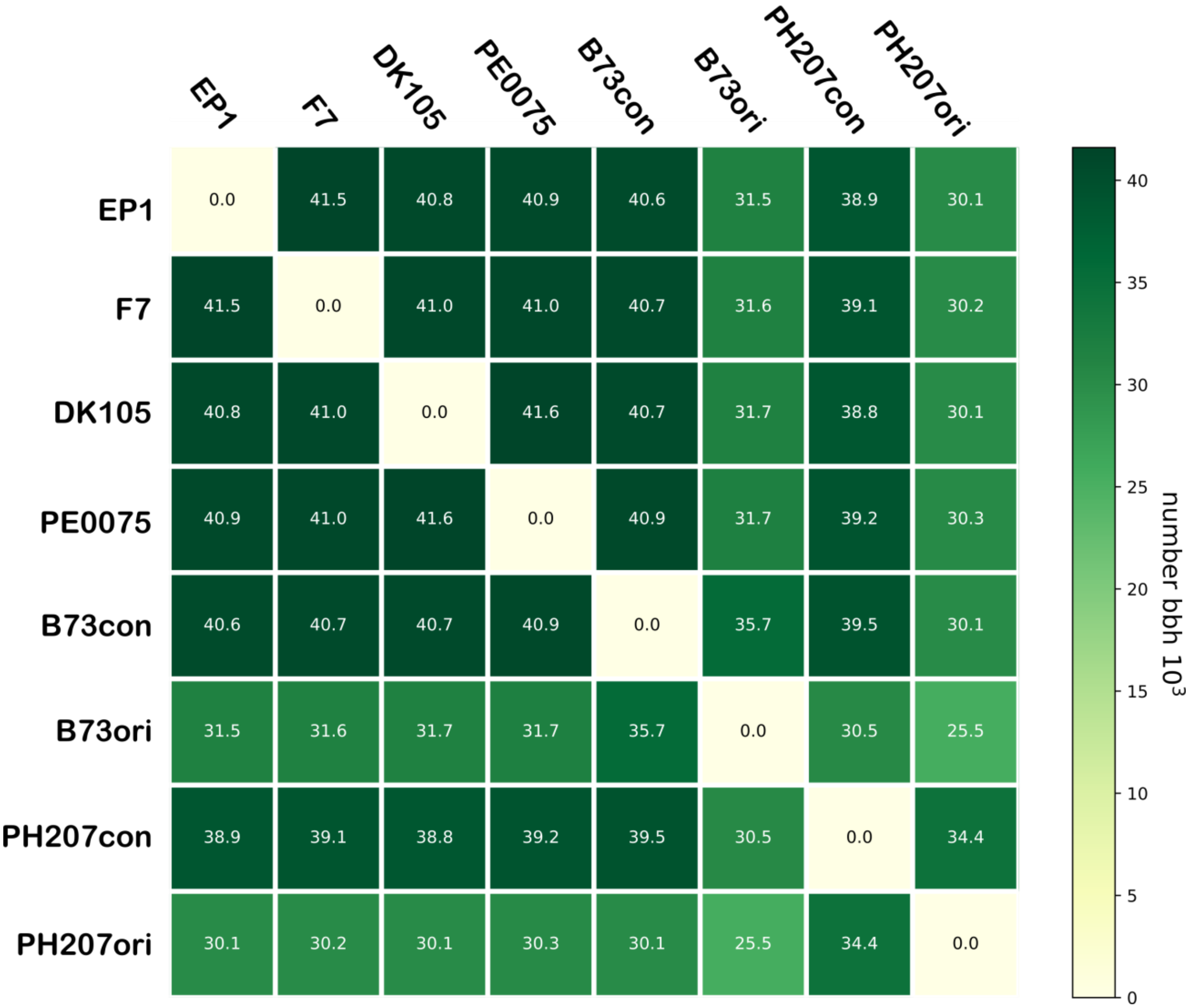

Supplementary Table 7. Repeat composition of eight maize lines. (a) Transposons detected via homology to REdat_9.8_Panicoideae, without overlapping annotations and as percent of the respective assembly length without Ns. Overall and subgroup numbers are very similar between all lines. Even PH207 is not much different in its transposon content, despite its lower assembly assembly quality. **(b)** Simple sequence tandem repeats and subgroups in Mb (overlaps removed). The large up to 15 fold differences in satellite and knob tandem repeats reflect different assembly strategies and not biological differences as show by a fish analyses.

a

(% of Nfree assembly)	PE0075	DK105	F7	EP1	B73v4	Mo17	W22	PH207
**Mobile Element (TXX)**	**80.0**	**80.4**	**80.7**	**81.1**	**79.5**	**79.4**	**79.7**	**77.2**
**Class I: Retroelement (RXX)**	**77.8**	**78.3**	**78.6**	**79.1**	**77.2**	**77.2**	**77.4**	**74.9**
LTR Retrotransposon (RLX)	77.5	77.9	78.3	78.8	76.8	76.8	77.1	74.5
Ty1/copia (RLC)	22.8	22.8	24.0	24.6	22.4	22.6	22.7	18.4
Ty3/gypsy (RLG)	37.4	37.6	36.7	36.7	37.4	37.3	37.1	36.3
unclassified LTR (RLX)	17.3	17.5	17.6	17.5	17.0	16.9	17.3	19.8
non-LTR Retrotransposon (RXX)	0.36	0.35	0.34	0.32	0.37	0.36	0.36	0.44
LINE (RIX)	0.36	0.35	0.34	0.32	0.37	0.36	0.36	0.44
**Class II: DNA Transposon (DXX)**	**2.16**	**2.11**	**2.07**	**2.03**	**2.28**	**2.22**	**2.20**	**2.29**
DNA Transposon Superfamily (DTX)	1.26	1.23	1.21	1.20	1.34	1.32	1.29	1.19
CACTA superfamily (DTC)	0.93	0.92	0.91	0.92	1.00	0.99	0.96	0.81
hAT superfamily (DTA)	0.09	0.09	0.09	0.09	0.10	0.09	0.10	0.11
Mutator superfamily (DTM)	0.11	0.11	0.11	0.10	0.12	0.12	0.11	0.11
Tc1/Mariner superfamily (DTT)	0.001	0.001	0.001	0.001	0.001	0.001	0.001	0.001
PIF/Harbinger (DTH)	0.11	0.10	0.10	0.09	0.11	0.11	0.11	0.14
unclassified (DTX)	0.01	0.01	0.01	0.01	0.01	0.01	0.01	0.01
MITE (DXX)	0.53	0.51	0.49	0.48	0.55	0.55	0.55	0.68
Helitron (DHH)	0.17	0.17	0.15	0.16	0.18	0.17	0.17	0.23
unclassified DNA transposon (DXX)	0.20	0.20	0.21	0.19	0.20	0.21	0.21	0.20
Retro-TE/DNA-TE ratio	*36.0*	*37.2*	*38.1*	*38.9*	*33.9*	*34.7*	*35.1*	*32.7*
Gypsy/Copia ratio	*1.6*	*1.6*	*1.5*	*1.5*	*1.7*	*1.7*	*1.6*	*2.0*

b

(Mb)	PE0075	DK105	F7	EP1	B73v4	Mo17	W22	PH207
Tandem repeats	81.7	93.8	109.0	121.2	67.4	79.1	65.7	50.5
Microsatellite (2-9bp units)	1.4	1.3	1.5	1.6	1.5	1.4	1.3	0.8
Minisatellite (10-99 units)	51.3	52.8	56.2	57.6	49.7	50.2	47.1	34.3
Satellite (>=100 bp units)	28.9	39.7	51.3	62.0	16.3	27.4	17.3	15.4
knob	9.61	14.40	27.29	29.83	2.62	9.12	1.89	4.06

Supplementary Table 8. Characteristics of *de novo* detected full length LTR retrotransposons in eight maize lines. (**a)** Main detection metrics. (**b)** Percent of shared syntenic locations for still intact full length elements, per superfamily and overall. Almost half of all fl-LTR locations are unique to one line. These structural differences are caused by very recent line specific insertions as well as by line specific removals or truncations and may additionally be biased by differing assembly approaches.

a

	line	candidates	quality filtered	% of candidates	RLC	RLG	RLX
flint	**PE0075**	80,501	14,827	18.4	2,873	4,970	6,984
	**DK105**	81,327	14,489	17.8	2,832	4,788	6,869
	**F7**	83,983	14,535	17.3	2,698	4,656	7,181
	**EP1**	85,072	14,946	17.6	2,862	4,697	7,387
dent	**B73v4**	78,275	14,777	18.9	3,020	5,068	6,689
	**Mo17**	79,524	14,181	17.8	3,000	5,200	5,981
	**W22**	78,871	12,977	16.5	2,709	4,194	6,074
	**PH207**	59,529	6,838	11.5	1,141	2,162	3,535

b

	less stringently filtered			high quality filtered		
shared between	number	percent of all	median age	number	percent of all	median age
**all**	540,050	100	0.68	106,826	100	0.74
**unique to 1 line**	224,717	41.6	0.54	43,660	40.9	0.58
**2 lines**	100,270	18.6	0.64	20,610	19.3	0.77
**3 lines**	72,111	13.4	0.75	14,532	13.6	0.84
**4 lines**	50,076	9.3	0.82	9,884	9.3	0.83
**5 lines**	35,815	6.6	0.92	7,245	6.8	0.92
**6 lines**	26,604	4.9	0.94	5,160	4.8	0.96
**7 lines**	18,977	3.5	1.00	3,815	3.6	0.97
**8 lines**	11,480	2.1	1.21	1,920	1.8	1.04

Supplementary Table 9. Total SAB sizes of the pairwise alignments of the six lines. Rows indicate the target genome line, columns the source genome aligned to the target. For example, the total size of SABs in the PH207 genome that has been detected by B73 (v4) genomic sequences is 1062 Mb. The highest proportion of aligned genomic regions are observed between lines of the same germplasm. Total SAB spans including PH207 are generally decreased due to the significantly larger gap sequences of this line. Note that total sizes can be slightly asymmetric due to differences in alignment gaps.

	B73v4	PH207	EP1	F7	DK105	PE0075
B73v4	0	1063	1156	1182	1177	1200
PH207	1062	0	970	973	948	1003
EP1	1156	970	0	1282	1246	1225
F7	1182	973	1282	0	1291	1241
DK105	1177	948	1246	1291	0	1275
PE0075	1200	1003	1225	1241	1275	0

Supplementary Table 10. Total sizes of pairwise MABs, see legend Supplementary Table 7 for details.

	B73v4	PH207	EP1	F7	DK105	PE0075
B73v4	0	1838	1802	1807	1798	1805
PH207	1742	0	1695	1706	1676	1708
EP1	1955	1933	0	2013	1998	1981
F7	1924	1905	1973	0	1967	1942
DK105	1893	1865	1934	1955	0	1940
PE0075	1834	1837	1860	1863	1874	0

Supplementary Table 11. Number of SNPs and total genomic size of the 31 higher order haplotypes. Group1 and group2 show the line combinations for which the haplotype is identical within each group and different between the groups. Further descriptions are provided in the main text and methods section.

Group 1	Group 2	SNP count	Size [bp]
B73v4	DK105;EP1;F7;PE0075;PH207	114041	31530980
B73v4;DK105;F7;PE0075;PH207	EP1	101909	24354517
B73v4;DK105;EP1;F7;PE0075	PH207	90947	21225493
B73v4;DK105;EP1;PE0075;PH207	F7	86032	23546344
B73v4;DK105;EP1;F7;PH207	PE0075	81117	20572261
B73v4;EP1;F7;PE0075;PH207	DK105	71501	18687278
**B73v4;PH207**	**DK105;EP1;F7;PE0075**	**71087**	**18763719**
B73v4;PE0075;PH207	DK105;EP1;F7	54276	14924194
B73v4;F7;PE0075;PH207	DK105;EP1	42024	9423938
B73v4;EP1;PE0075;PH207	DK105;F7	38763	9487100
B73v4;EP1;PH207	DK105;F7;PE0075	28113	6723233
B73v4;DK105;EP1;F7	PE0075;PH207	26926	6972824
B73v4;F7;PH207	DK105;EP1;PE0075	26594	9456847
B73v4;DK105	EP1;F7;PE0075;PH207	24046	6517900
B73v4;DK105;EP1;PE0075	F7;PH207	23052	4879837
B73v4;EP1;F7;PH207	DK105;PE0075	22231	6282974
B73v4;DK105;PE0075;PH207	EP1;F7	20518	5071033
B73v4;DK105;F7;PE0075	EP1;PH207	20513	4213886
B73v4;DK105;EP1;PH207	F7;PE0075	19468	5462675
B73v4;DK105;F7;PH207	EP1;PE0075	18091	4060362
B73v4;DK105;PH207	EP1;F7;PE0075	16329	4362025
B73v4;PE0075	DK105;EP1;F7;PH207	16251	5553633
B73v4;DK105;EP1	F7;PE0075;PH207	14082	2937247
B73v4;EP1;PE0075	DK105;F7;PH207	12244	4088402
B73v4;F7	DK105;EP1;PE0075;PH207	12078	2811297
B73v4;EP1	DK105;F7;PE0075;PH207	11023	2804651
B73v4;DK105;PE0075	EP1;F7;PH207	10456	2645995
B73v4;F7;PE0075	DK105;EP1;PH207	10402	3669615
B73v4;DK105;F7	EP1;PE0075;PH207	9862	2362083
B73v4;EP1;F7;PE0075	DK105;PH207	8412	2112371
B73v4;EP1;F7	DK105;PE0075;PH207	8041	2639432
		**1110429**	**288144146**

Supplementary Table 12. RNAseq quality summary. Raw and clean bases are denoted by Gb.

Sample	Raw Reads	Clean Reads	Raw bases	Clean bases	Effective rate (%)	Error rate (%)	Q20 (%)	Q30 (%)	GC content (%)
**F7**	688499867	667448978	206.55	200.23	96.94	0.02	95.31	89.19	56.60
**EP1**	692842987	652393710	207.85	195.72	94.16	0.01	95.92	90.25	56.45

Supplementary Figures

Supplementary Figure 1. Genome size estimation by kmer spectra. (a) shows kmer spectra obtained by Genomescope2 (kmer size K=21 bp, maximal count limit = 10,000) for the 4 European flint lines of this study. **(b)** Table rows list in [bp] from top to bottom the genome size estimated by kmer spectra, the assembled size for the pseudochromosomes and the total assembly size including unanchored scaffolds. Total assembly sizes of EP1 and F7 slightly exceed estimated genome sizes, in particular due to unanchored scaffold sequences, which may contain assembly path duplications.

a

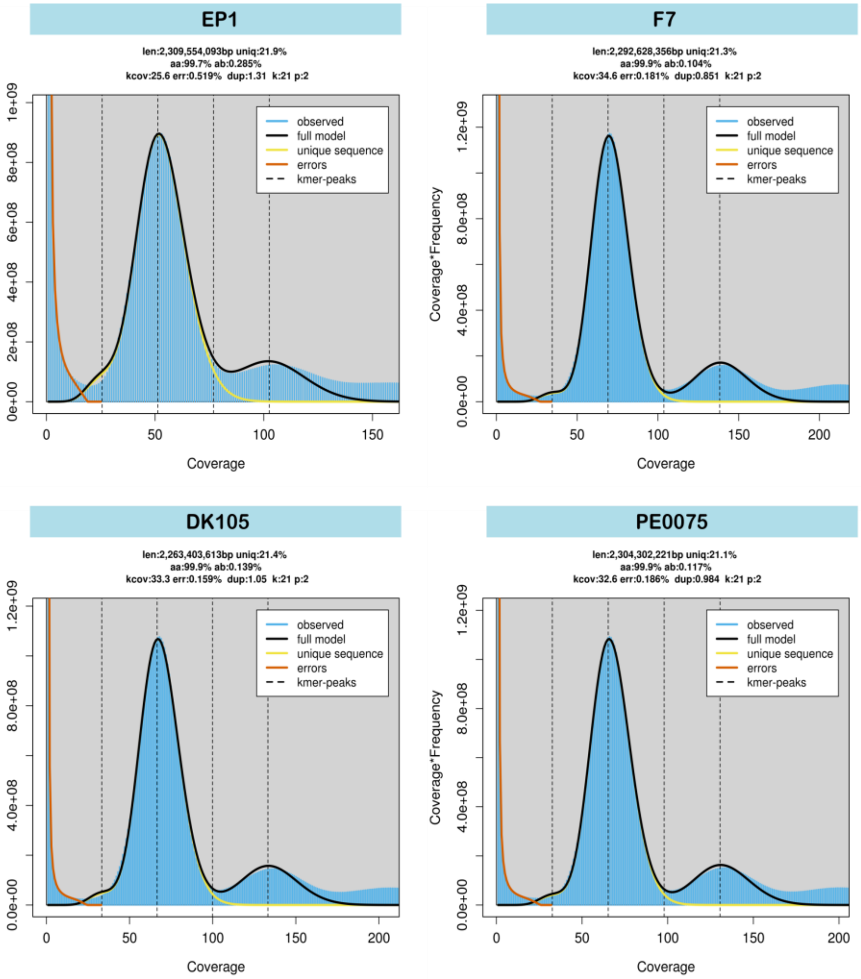

b

	DK105	EP1	F7	PE0075
**Kmer estimate**	2,263,403,613	2,309,554,093	2,292,628,356	2,304,302,221
**pseudomolecules**	2,176,062,556	2,321,039,442	2,255,461,729	2,140,670,148
**Total assembly**	2,288,189,157	2,455,259,639	2,392,801,755	2,198,504,211

Supplementary Figure 2. Orhologous cluster of six maize lines. Bar chart displays number of orthologous clusters (y-axis) derived from bi-directional best blast hits of the six maize lines B73, PH207, EP1, F7, DK105 and PE0075 and subdivided by the number of distinct maize lines contributing to an orthologous group (x-axis). Results obtained using the original versus consolidated B73 and PH207 annotations are shown as yellow and blue columns, respectively.

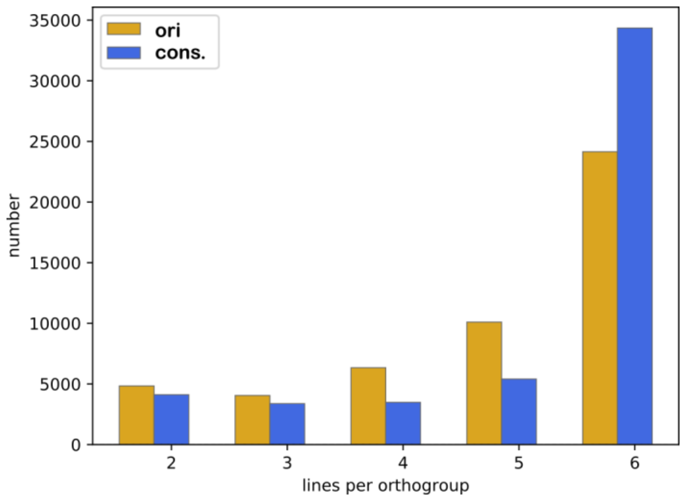

Supplementary Figure 3. Net change of the original to the consolidated gene annotations in B73 and PH207. Waterfall charts show the number of identical, overlapping (shared) and unique gene models between the original (ori) and consolidated (new) gene annotations for B73 **(a)** and PH207 **(b)**, respectively. Differences for the 'ori' and 'new' shared gene numbers result from merged and split gene models. Several thousand models unique to the original annotations show expression in a series of major tissue RNAseq data. **Expression levels of unique models for B73 (c) and PH207 (d).** The lower panel shows the number of expressed genes in dependency of the applied TPM threshold (x-axis). Expression data are based on NCBI Bioprojects PRNJA357594, PRNJA376191, PRNJA477253, PRNJA482146, PRNJA494874, PRNJA507752, PRNJA511671, PRNJA524898, PRNJA532439, PRNJA548548 for B73; and PRNJA258455, PRNJA385873, PRNJA562045 for PH207.

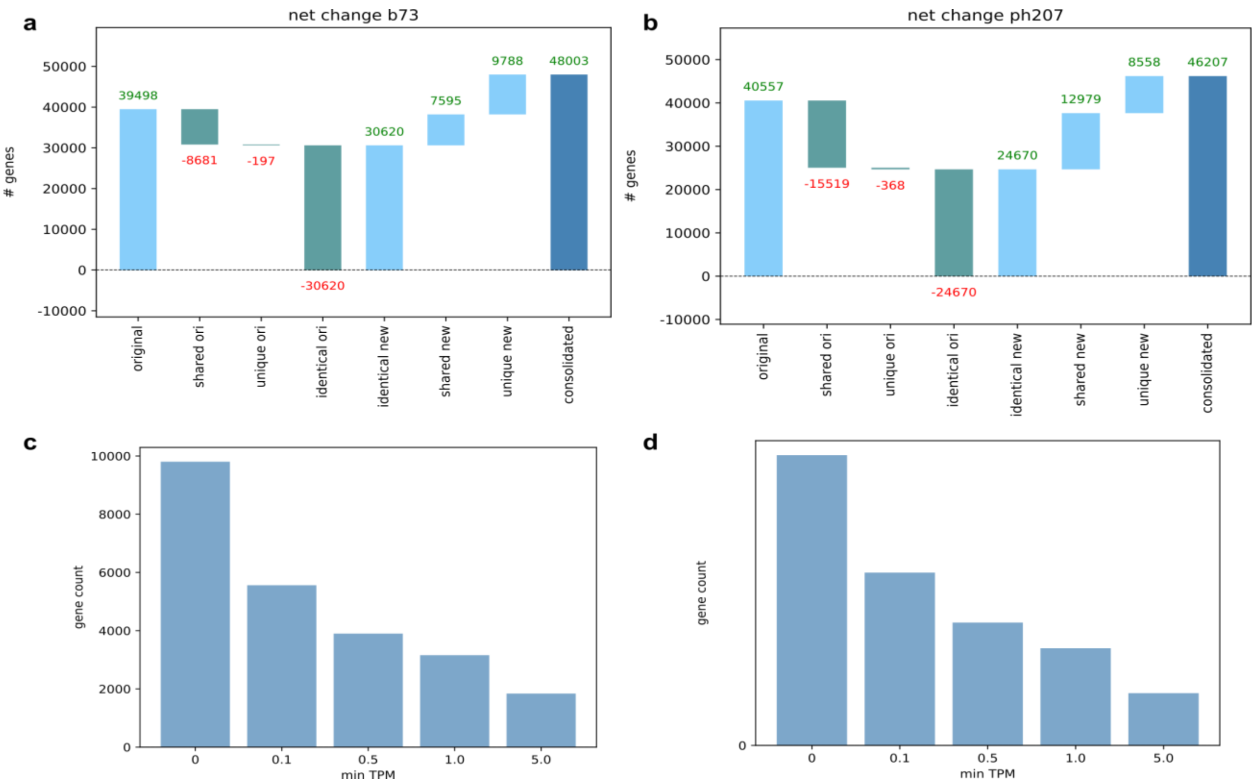

Supplementary Figure 4. Chromosomal distribution of satellite tandem repeats in the assemblies of eight maize lines. The four NRGene assemblies contain larger amounts of satellite tandem repeats compared to the B73 and PH207 assemblies (Figure 2B). For EP7 they have even been placed in their correct chromosomal context on chromosomes 4L, 5L, and 6S as proven by the fish data (Figure 2a). For DK105 and PE0075 the 5L and 6S locations are present in the assembly, but not the prominent 9S location. Most of the highly repetitive satellite sequences could not be assigned to a specific chromosomal position. They have been merged into the unassigned sequence pool (Un).

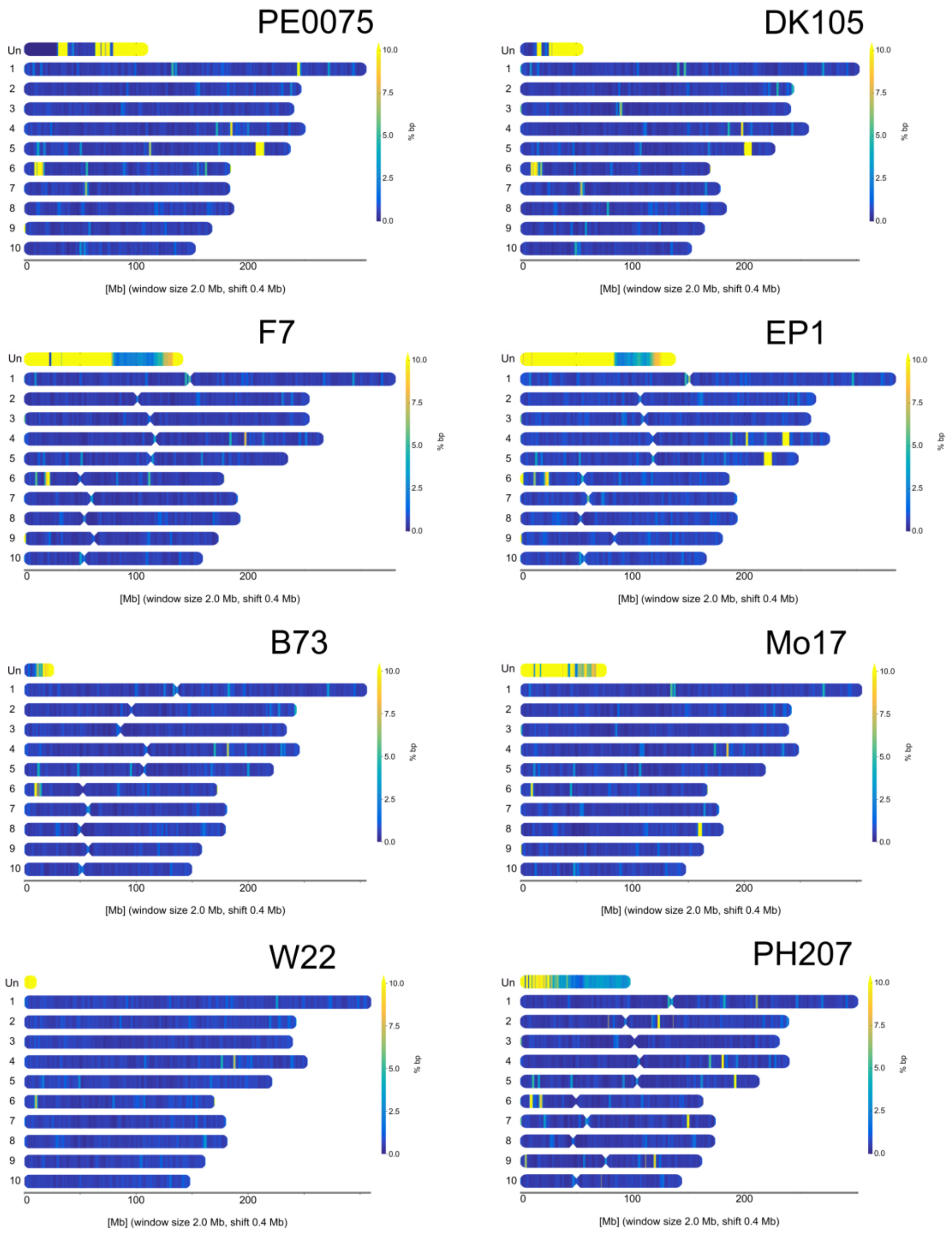

Supplementary Figure 5. Sketch illustrating the chaining of single alignment blocks (SABs) to merged alignment block (MABs). SABs were computed as global (1:1) orthologous blocks using the MUMMER tool (see methods). MABs are chains of consistent SABs (upper panel) which can be linked by no edit operation (e.g. insertion, translocation, inversion). The lower panel provides examples of non-permissive edit operations.

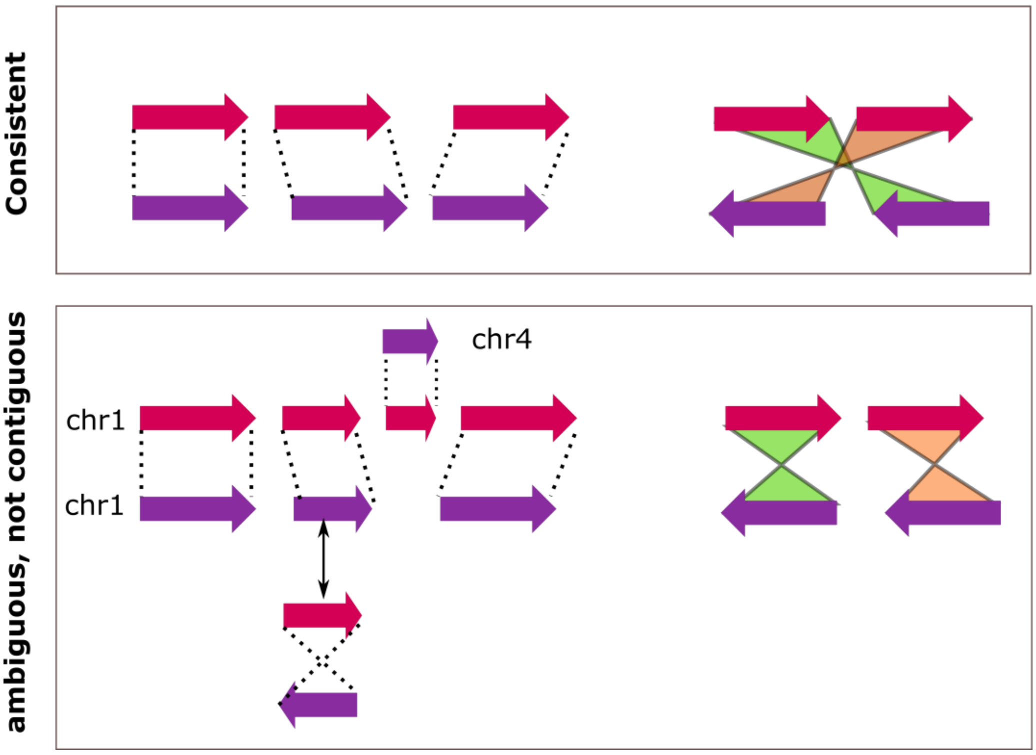

Supplementary Figure 6. SAB and MAB sizes for all 15 pairwise WGA comparisons of the six maize lines EP1, F7, DK105, PE0075, B73 (version 4) and PH207. Mean and median are indicated in the boxplot by an asterisk and yellow line, respectively.

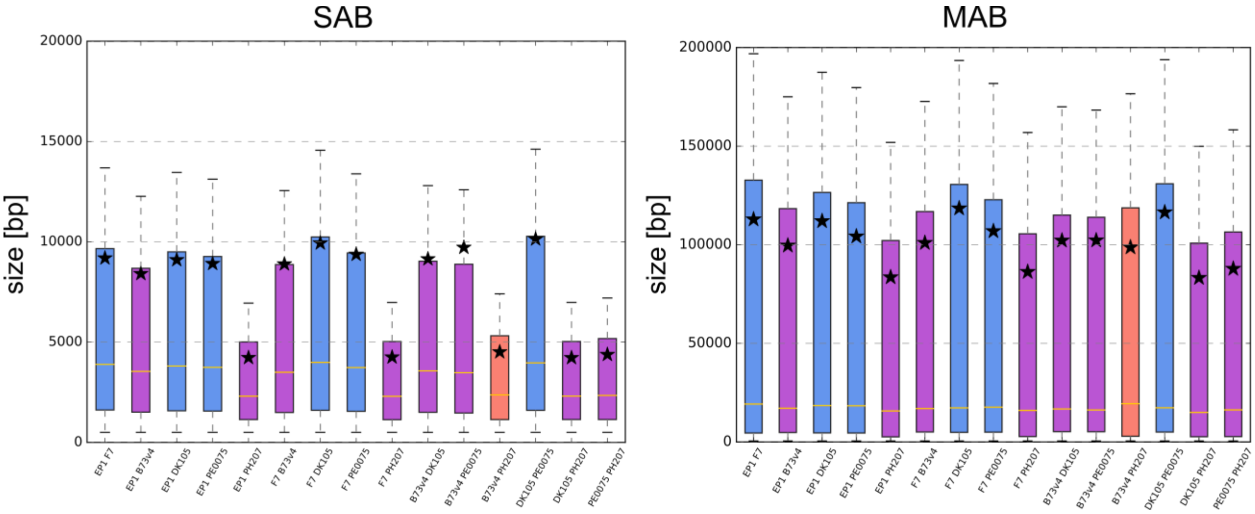

Supplementary Figure 7. Orthology support of non-inverted WGA blocks by read cross-mapping. Genomic Illumina read pairs (insert size 400 bp) of F7 and DK105 were mapped to both assemblies using BWA. For each segment of the pairwise WGA of F7-DK105 with a parallel alignment orientation (i.e. excluding inversions), read identifiers were tracked to determine the fraction of reads (x-axis, '%agreement') consistently mapping to both respective regions. Median overlap between F7 and DK105 reads was ~93%. Additional combinations of line pairs (inlet plot) showed equally high consistencies supporting the orthology of parallel WGA blocks.

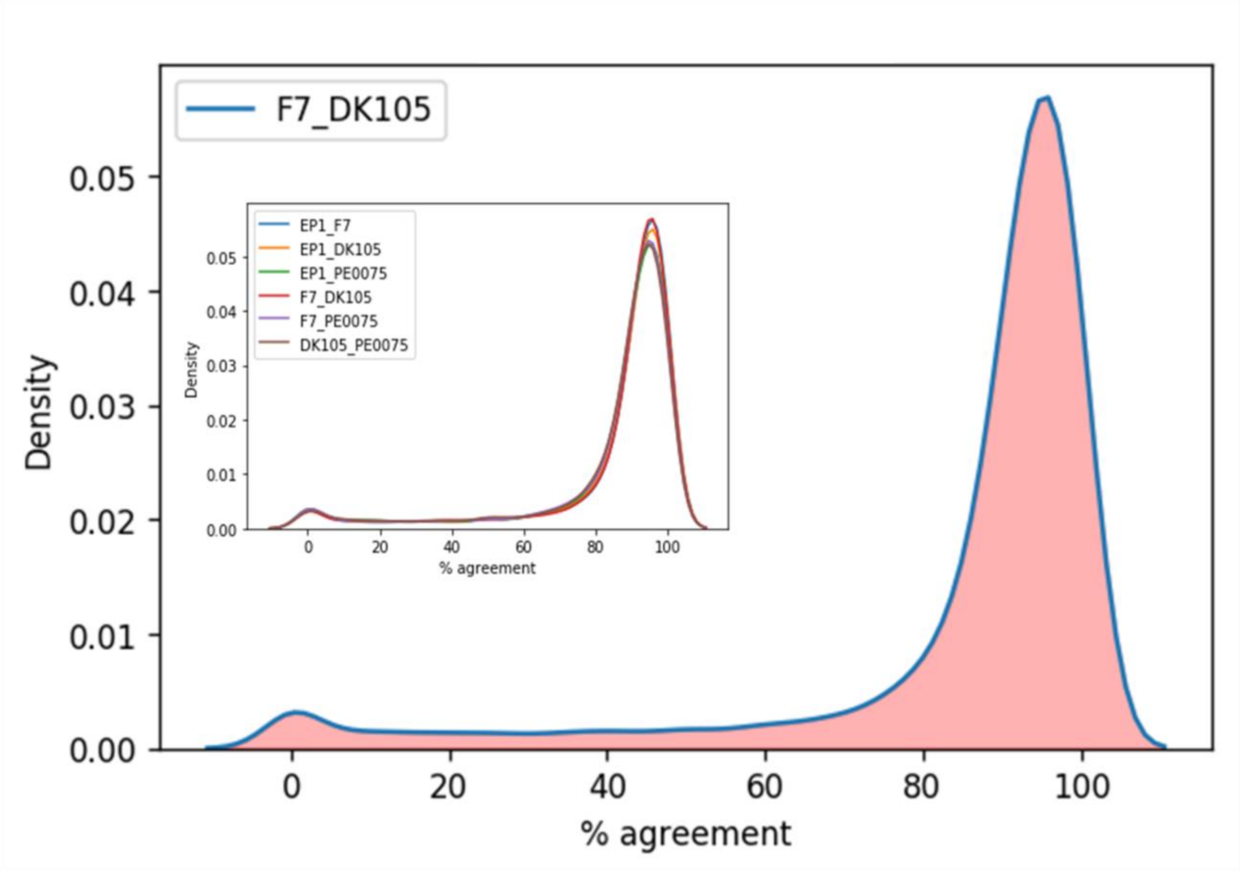

Supplementary Figure 8. Concept of the genomic core-, group-specific and unaligned regions generated from pairwise WGAs. Pairwise alignments of EP1 with B73, PH207, F7, DK105 and PE0075 are projected onto the EP1 sequence providing a uniform coordinate system. Such a projection can be performed for MABs (upper panel) and SABs (lower panel). Regions delineated as unaligned regions, group-specific and core regions are labeled 1, 2 and 3, respectively. Note that labeling can substantially vary between MABs or SABs due to the ability of MABs to span unaligned regions between two SABs as long as they can be contiguously linked (see Supplementary Figure 6).

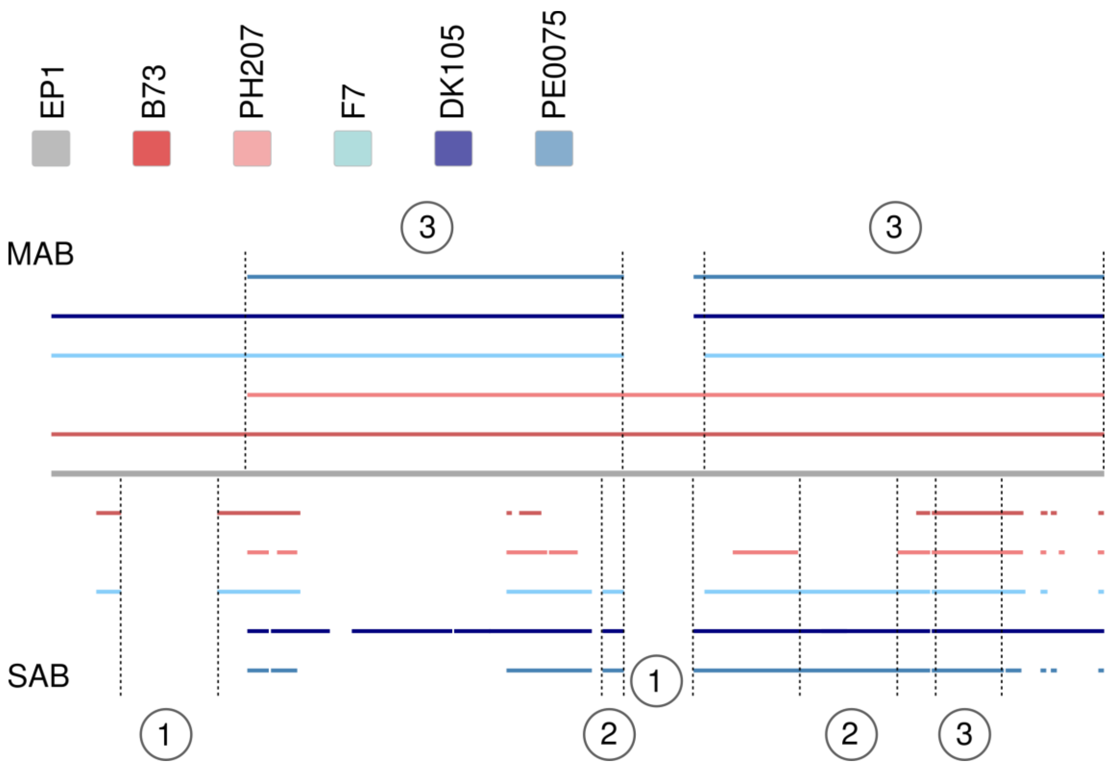

Supplementary Figure 9. Lines selected for the European flint-dent haplotype expression analysis. Hapmap v3 SNPs were restricted to the genomic regions defined by the haplotype differentiating European flint lines and dent lines. Based on these variation data, a phylogenetic analysis detected NAM maize lines closely related to B73 (red) or EP1/F7 (blue) which were subsequently analyzed for differential expression. For readability, the tree only shows the subtree of the NAM panel most relevant for the line selection.

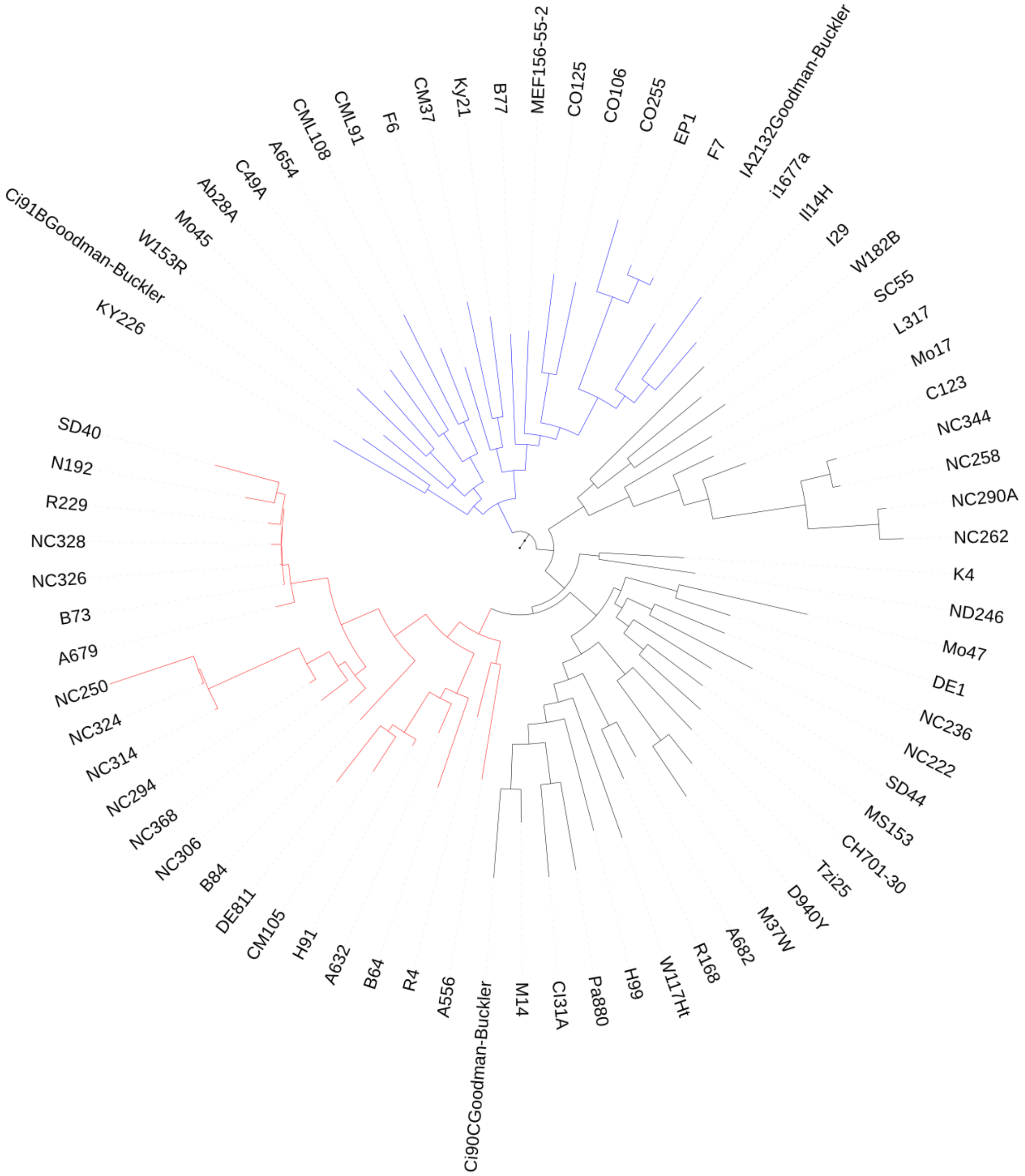

Supplementary Figure 10. Support for estimated gap sizes of sequence stretches of undefined bases (‘N’) in the four flint assemblies. Panel (**a**) shows the histogram of the sizes of undefined sequences (‘N’; gaps) inserted into DK105 assemblies. A large majority of gaps are less than 1kb long. To derive upper estimates for assembly gaps, genomic reads of several libraries with insert sizes 450 bp, 800 bp, 2 kb, 5 kb and 8 kb were aligned to the genome (see Supplementary Table 1) and a minimum of 10 mates mapping up- and downstream of the undefined region were required for a gap to be to counted as covered. (**b**) tabulates the number of total gaps binned by size ranges. Gap coverage for each bin and library as well as uncovered gaps are shown in column 3-8 relative to the bin total. Coverage is cumulative, i.e. only additional gaps not yet covered by libraries with lower insert sizes are listed. Panel (**c**) illustrates the proportion of gaps cumulatively covered by five libraries with increasing insert sizes in F7, EP1, PE0075 and DK105. One square represents ~1% of the total gap count in each line, uncovered gaps are shown in yellow.

a

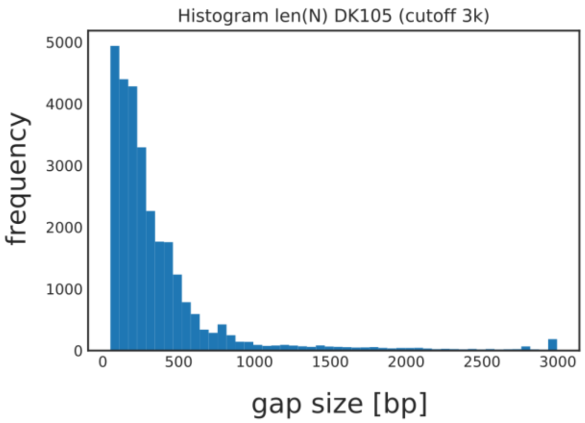

b

Interval [bp]	Total	450bp	800bp	2kbp	5kbp	8kbp	No cov
≤500	23540	44%	21%	31%	1%	0%	4%
]500,1000]	3361	8%	5%	84%	1%	0%	1%
]1000,3000]	1703	9%	6%	76%	9%	1%	0%
]3000,7000]	688	11%	7%	27%	47%	8%	0%
>7000	47	13%	0%	13%	21%	45%	9%

c

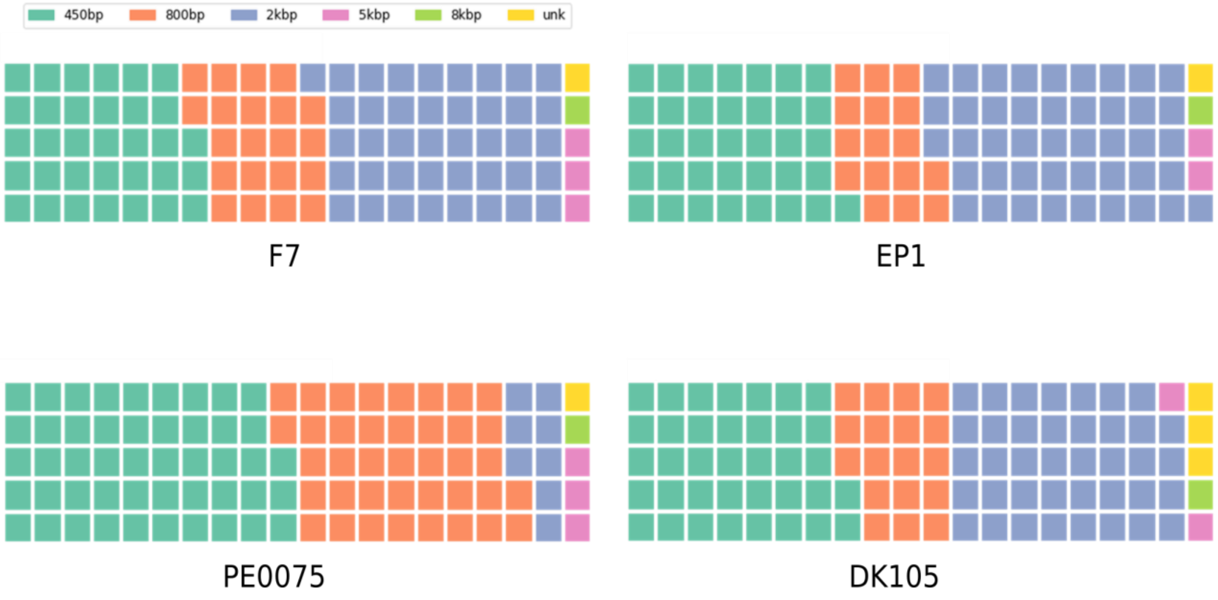

Supplementary Figure 11. Expression levels (TPM; y-axis) for four distinct confidence gene classes (high confidence: HC; low confidence 1 to 3: LC1-3). Flyers show 1.5 quartile range, white central line mean TPM values.

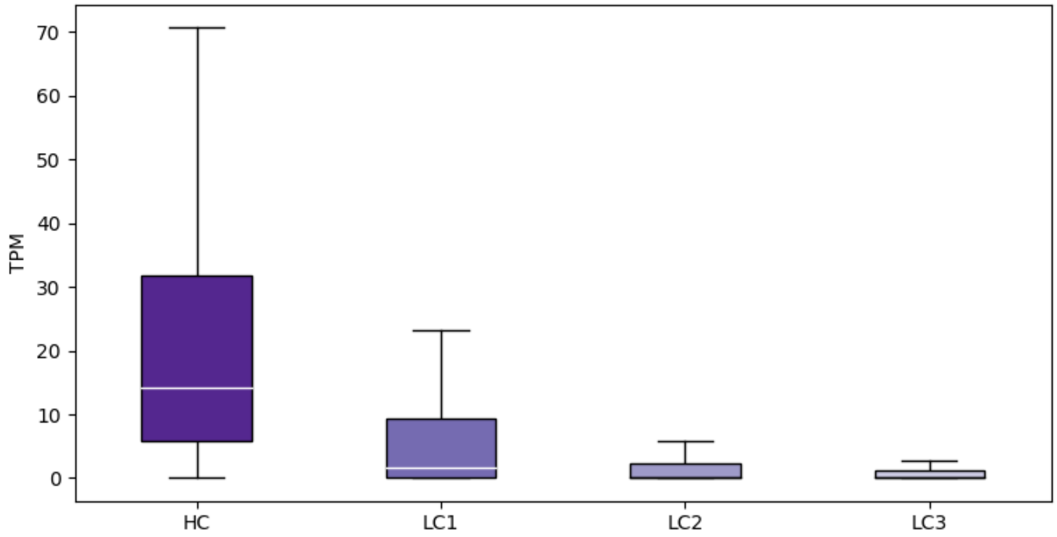

Supplement: Supplementary file 1 — Supplementary Notes, Tables 1–12 and Figs. 1–11 [file 41588_2020_671_MOESM1_ESM.pdf]
