# Supplementary material for: European maize genomes highlight intraspecies variation in repeat and gene content
Source: Nat Genet. 2020 Jul 27;52(9):950–7. doi: 10.1038/s41588-020-0671-9 (PMC7467862; doi:10.1038/s41588-020-0671-9)
Supplement: Supplementary file 2 — Reporting Summary [file 41588_2020_671_MOESM2_ESM.pdf]

nature research

Corresponding author(s): Klaus FX Mayer
Chris Carolin Schoen

Last updated by author(s): May 29, 2020

Reporting Summary

Nature Research wishes to improve the reproducibility of the work that we publish. This form provides structure for consistency and transparency in reporting. For further information on Nature Research policies, see Authors & Referees and the Editorial Policy Checklist.

Statistics

For all statistical analyses, confirm that the following items are present in the figure legend, table legend, main text, or Methods section.

n/a	Confirmed	
☐	☒	The exact sample size (*n*) for each experimental group/condition, given as a discrete number and unit of measurement
☐	☒	A statement on whether measurements were taken from distinct samples or whether the same sample was measured repeatedly
☐	☒	The statistical test(s) used AND whether they are one- or two-sided<br>*Only common tests should be described solely by name; describe more complex techniques in the Methods section.*
☐	☒	A description of all covariates tested
☐	☒	A description of any assumptions or corrections, such as tests of normality and adjustment for multiple comparisons
☐	☒	A full description of the statistical parameters including central tendency (e.g. means) or other basic estimates (e.g. regression coefficient) AND variation (e.g. standard deviation) or associated estimates of uncertainty (e.g. confidence intervals)
☐	☒	For null hypothesis testing, the test statistic (e.g. *F*, *t*, *r*) with confidence intervals, effect sizes, degrees of freedom and *P* value noted<br>*Give P values as exact values whenever suitable.*
☒	☐	For Bayesian analysis, information on the choice of priors and Markov chain Monte Carlo settings
☒	☐	For hierarchical and complex designs, identification of the appropriate level for tests and full reporting of outcomes
☐	☒	Estimates of effect sizes (e.g. Cohen's *d*, Pearson's *r*), indicating how they were calculated

Our web collection on statistics for biologists contains articles on many of the points above.

Software and code

Policy information about availability of computer code

Data collection	Illumina HiSeq 2500 and 4000 produced genomic and RNAseq paired-end reads, respectively;
Data analysis	Mummer v3.23; blat v34; ncbi-blast+ 2.2.28+; exonerate 2.2.0-x86 64 bit version; fast sequence aligner fsa-1.15.9; GenomeThreader v1.6.4; bwa 0.7.17; STAR v2.5.1a; vmatch 2.3.0; samtools v1.3; bcftools v1.3; vcftools v0.1.15; HTseq 0.11.2; R packages ASMap v. 1.0-2, DESeq2 v1.23.3; Trinity 2.4.0; Bridger version r2014-12-01 ; TandemRepeatFinder 4.09; genometools (including LTRharvest, LTRdigest & Tallymer) gt-1.5.3; hmmer3; LTR retriever (LTR Finder within retriever suite); fasttree v2.1.5 SSE3; bbmap v38.31; custom python and R scripts/code will be made available upon request

For manuscripts utilizing custom algorithms or software that are central to the research but not yet described in published literature, software must be made available to editors/reviewers. We strongly encourage code deposition in a community repository (e.g. GitHub). See the Nature Research guidelines for submitting code & software for further information.

Data

Policy information about availability of data

All manuscripts must include a data availability statement. This statement should provide the following information, where applicable:

- Accession codes, unique identifiers, or web links for publicly available datasets
- A list of figures that have associated raw data
- A description of any restrictions on data availability

Data has been released as NCBI bioproject PRJNA360923 and PRJNA360920. Genome sequences and gene models (GFFs) are also available at maizeGDB (https://www.maizegdb.org)

Field-specific reporting

Please select the one below that is the best fit for your research. If you are not sure, read the appropriate sections before making your selection.

☒ Life sciences ☐ Behavioural & social sciences ☐ Ecological, evolutionary & environmental sciences

For a reference copy of the document with all sections, see nature.com/documents/nr-reporting-summary-flat.pdf

Life sciences study design

All studies must disclose on these points even when the disclosure is negative.

Sample size	Sequence read depths followed recommendations of NRGene's DeNovoMagick assembly pipeline
Data exclusions	No data excluded
Replication	No replication required/meaningful for study
Randomization	Not applicable
Blinding	Not applicable

Reporting for specific materials, systems and methods

We require information from authors about some types of materials, experimental systems and methods used in many studies. Here, indicate whether each material, system or method listed is relevant to your study. If you are not sure if a list item applies to your research, read the appropriate section before selecting a response.

Materials & experimental systems

n/a	Involved in the study	
☒	☐	Antibodies
☒	☐	Eukaryotic cell lines
☒	☐	Palaeontology
☐	☒	Animals and other organisms
☒	☐	Human research participants
☒	☐	Clinical data

Methods

n/a	Involved in the study	
☒	☐	ChIP-seq
☒	☐	Flow cytometry
☒	☐	MRI-based neuroimaging

Animals and other organisms

Policy information about studies involving animals; ARRIVE guidelines recommended for reporting animal research

Laboratory animals	Maize (Zea mays ssp. mays) lines EP1, F7, DK105, PE0075 (doubled haploid line from Petkuser population)
Wild animals	not applicable
Field-collected samples	not applicable
Ethics oversight	no ethical guidance required

Note that full information on the approval of the study protocol must also be provided in the manuscript.
